# Epilepsy-associated gene *Nedd4-2* mediates neuronal activity and seizure susceptibility through AMPA receptors

**DOI:** 10.1371/journal.pgen.1006634

**Published:** 2017-02-17

**Authors:** Jiuhe Zhu, Kwan Young Lee, Kathryn A. Jewett, Heng-Ye Man, Hee Jung Chung, Nien-Pei Tsai

**Affiliations:** 1 Department of Molecular and Integrative Physiology, School of Molecular and Cellular Biology, University of Illinois at Urbana-Champaign, Urbana, IL, United States of America; 2 Department of Biology, Boston University, Boston, MA, United States of America; 3 Department of Pharmacology and Experimental Therapeutics, Boston University School of Medicine, Boston, MA, United States of America; 4 Neuroscience Program, University of Illinois at Urbana-Champaign, Urbana, IL 61801, United States of America; 5 Beckman Institute for Advanced Science and Technology, University of Illinois at Urbana-Champaign, Urbana, IL, United States of America; Columbia University Medical Center, UNITED STATES

## Abstract

The neural precursor cell expressed developmentally down-regulated gene 4–2, *Nedd4-2*, is an epilepsy-associated gene with at least three missense mutations identified in epileptic patients. *Nedd4-2* encodes a ubiquitin E3 ligase that has high affinity toward binding and ubiquitinating membrane proteins. It is currently unknown how *Nedd4-2* mediates neuronal circuit activity and how its dysfunction leads to seizures or epilepsies. In this study, we provide evidence to show that *Nedd4-2* mediates neuronal activity and seizure susceptibility through ubiquitination of GluA1 subunit of the α-amino-3-hydroxy-5-methyl-4-isoxazolepropionic acid receptor, (AMPAR). Using a mouse model, termed *Nedd4-2*^*andi*^, in which one of the major forms of *Nedd4-2* in the brain is selectively deficient, we found that the spontaneous neuronal activity in *Nedd4-2*^*andi*^ cortical neuron cultures, measured by a multiunit extracellular electrophysiology system, was basally elevated, less responsive to AMPAR activation, and much more sensitive to AMPAR blockade when compared with wild-type cultures. When performing kainic acid-induced seizures *in vivo*, we showed that elevated seizure susceptibility in *Nedd4-2*^*andi*^ mice was normalized when GluA1 is genetically reduced. Furthermore, when studying epilepsy-associated missense mutations of *Nedd4-2*, we found that all three mutations disrupt the ubiquitination of GluA1 and fail to reduce surface GluA1 and spontaneous neuronal activity when compared with wild-type Nedd4-2. Collectively, our data suggest that impaired GluA1 ubiquitination contributes to Nedd4-2-dependent neuronal hyperactivity and seizures. Our findings provide critical information to the future development of therapeutic strategies for patients who carry mutations of *Nedd4-2*.

## Author summary

Many patients with neurological disorders suffer from an imbalance in neuronal and circuit excitability and present with seizure or epilepsy as the common comorbidity. Human genetic studies have identified many epilepsy-associated genes, but the pathways by which those genes are connected to brain circuit excitability are largely unknown. Our study focused on one of the epilepsy-associated genes, *Nedd4-2*, and aimed to dissect the molecular mechanism underlying *Nedd4-2*-associated epilepsy. *Nedd4-2* encodes a ubiquitin E3 ligase. Several neuronal ion channels have been identified as its substrates, including the GluA1 subunit of AMPAR. Our results first demonstrate up-regulation of spontaneous neuronal activity and seizure susceptibility when *Nedd4-2* is reduced in a mouse model. These deficits can be corrected when GluA1/AMPAR is pharmacologically or genetically inhibited. In addition, we found that three epilepsy-associated missense mutations of *Nedd4-2* inhibit the ubiquitination of GluA1 and fail to reduce GluA1 surface expression or spontaneous neuronal activity when compared to wild-type *Nedd4-2*. These findings suggest the reduction of GluA1 ubiquitination as a crucial deficit underlying insufficient function of *Nedd4-2* and provide critical information to the development of therapies for patients who carry mutations of *Nedd4-2*.

## Introduction

A hyperactive brain circuit is a common abnormality observed in patients with various neurological and psychiatric disorders, including epilepsies (1). Evidence from human genetic studies implicates genes encoding ion channels or their regulators in the etiology of those pathophysiological conditions [1, 2]. Characterizing those genes and their function in regulation of brain circuit activity is likely to reveal novel therapeutic targets for these diseases. One of those genes is the neural precursor cell expressed developmentally downregulated gene 4-like (*Nedd4-2*) [3]. *Nedd4-2*, is an epilepsy-associated gene containing at least three missense mutations identified through genomic mutation screening in patients with epilepsy [3–6]. *Nedd4-2* encodes a ubiquitin E3 ligase that belongs to the Nedd4 family of ubiquitin E3 ligases [7] but is the only member encoded by an epilepsy-associated gene [3]. Because of an N-terminal lipid-binding domain, Nedd4-2 has high affinity toward binding and ubiquitinating membrane proteins [8]. Several neuronal membrane substrates of Nedd4-2 have been identified, such as voltage-gated sodium channel Na_v_1.6 [9], voltage-gated potassium channels K_v_7/KCNQ [10–12], neurotrophin receptor TrkA [13, 14] and the GluA1 subunit of the α-amino-3-hydroxy-5-methyl-4-isoxazolepropionic acid receptor (AMPAR) [15]. Our previous work has demonstrated elevated seizure susceptibility in mice when *Nedd4-2* is knocked down [16]. However, the mechanisms by which the dysfunction of Nedd4-2 contributes to epileptogenesis are unclear. Presumably wild-type (WT) Nedd4-2 mediates or represses circuit activity by ubiquitinating one or more of its substrates while the epilepsy-associated mutants fail to do so and lead to seizures and/or epilepsies. To test this possibility, we needed to identify the relevant substrate of Nedd4-2 in regulation of neuronal excitability and characterize the effect of epilepsy-associated mutations on substrate recognition.

The AMPAR is a major subtype of ionotropic glutamate receptors and is the most commonly found receptor in the mammalian nervous system [17, 18]. AMPARs are assembled as homo-or hetero-tetramers and are comprised of combinations of GluA1–GluA4 subunits [19]. Each subunit has a non-conserved C-terminal, an intracellular domain that harbors regulatory elements subject to various post-translational modifications such as ubiquitination. All four AMPAR subunits can be ubiquitinated, but only GluA1 ubiquitination has been specifically described upon different activity stimulations [20, 21]. Studies have shown that GluA1 ubiquitination contributes to its internalization [22, 23]. This internalization, part of AMPAR trafficking mechanisms [24], is critical for synaptic depression as well as homeostatic regulation of synaptic strength [25–28]. Because GluA1-GluA2 is the predominant AMPAR heteromer [29], and GluA1 is required for successful trafficking and targeting of GluA2 [30], we hypothesized that Nedd4-2 is required for limiting GluA1 surface expression and functionality of AMPAR. Because GluA1 levels affect neuronal activity [31], and dysregulation of AMPARs has been shown to be linked to epilepsy [32, 33], Nedd4-2 may play a role in affecting neuronal activity, seizures, and/or epilepsy through fine-tuning of AMPARs.

In our current study, we provide *in vitro* and *in vivo* evidence to demonstrate GluA1- and AMPAR-dependent elevation of neuronal activity and seizure susceptibility induced by functional insufficiency of Nedd4-2. To our knowledge, our findings provide the first mechanism underlying *Nedd4-2*-associated circuit hyperactivity and seizures and open up a new avenue for the development of therapeutic strategies to potentially treat epileptic patients who carry *Nedd4-2* mutations.

## Results

### Nedd4-2 mediates spontaneous neuronal and synaptic activity

**Fig 1:**
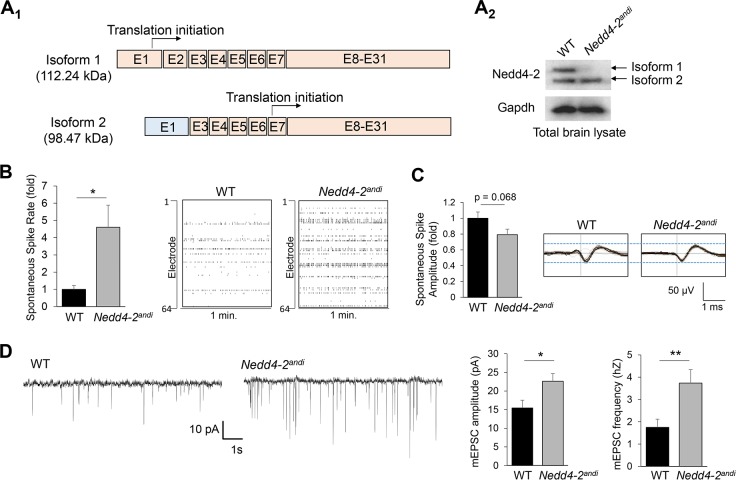
Nedd4-2 mediates spontaneous neuronal and synaptic activity. (**A**_**1**_) A diagram showing two alternative protein products (isoforms 1 and 2) from the *Nedd4-2* gene, and (**A**_**2**_) western blot results of Nedd4-2 and Gapdh from brain lysates of WT or *Nedd4-2*^*andi*^ mice at age 4-weeks old. (**B**) Quantification of spontaneous spike rate (left) and representative raster plots of spontaneous spikes from 1-min recording (right) of WT or *Nedd4-2*^*andi*^ cortical neuron cultures at DIV 13–14. (**C**) Quantification of average spontaneous spike amplitude (left) and representative average traces of 1-min recording (right) of WT or *Nedd4-2*^*andi*^ cortical neuron cultures at DIV 13–14. The black lines represent the average of all the spikes within representative 1-min recordings. (**D**) Patch-clamp recording from WT or *Nedd4-2*^*andi*^ cortical neurons at DIV 14. Representative mEPSC traces and quantification of mEPSC amplitude and frequency are shown (n = 15 for both WT or *Nedd4-2*^*andi*^ neurons). Data are analyzed by Student’s *t*-test and represented as mean ± SEM with *p<0.05, **p<0.01.

It is unknown how dysregulation of Nedd4-2 is involved in seizures or epilepsies. To answer this question, we employed a mouse model, *Nedd4-2*^*andi*^, in which the long-form (isoform 1) of Nedd4-2 is selectively deleted due to a spontaneous mutation in exon-2 (Fig 1A). Because *Nedd4-2* knockout mice are not viable [34], *Nedd4-2*^*andi*^ serves as an ideal, *in vivo* knockdown model to study Nedd4-2. To assess the question of whether Nedd4-2 mediates neuronal activity, we employed a multielectrode array (MEA) recording system (S1 Fig) to record extracellular spontaneous spikes of electrical activity in primary cortical neuron cultures prepared from WT or *Nedd4-2*^*andi*^ mice. As shown in Fig 1B, the frequency of spontaneous spikes was significantly elevated in *Nedd4-2*^*andi*^ cultures in comparison to WT cultures. The average spontaneous spike amplitude did not differ between WT and *Nedd4-2*^*andi*^ cultures (Fig 1C). These data indicate that spontaneous neuronal activity is basally elevated in *Nedd4-2*^*andi*^ cortical cultures.

To determine whether elevated spontaneous neuronal activity in *Nedd4-2*^*andi*^ cultures is accompanied by altered synaptic transmission, we performed whole-cell patch-clamp recording to obtain miniature excitatory post-synaptic current (mEPSC) from WT or *Nedd4-2*^*andi*^ cortical neuron cultures. As shown in Fig 1D, *Nedd4-2*^*andi*^ neurons exhibit elevation of both mEPSC amplitude and frequency when compared to WT neurons. These data suggest that Nedd4-2 likely mediates both pre- and post-synaptic properties, and the elevation of spontaneous neuronal activity observed in *Nedd4-2*^*andi*^ cortical cultures (Fig 1C) is likely contributed by multiple factors.

### Nedd4-2 mediates AMPAR-dependent spontaneous neuronal activity

**Fig 2:**
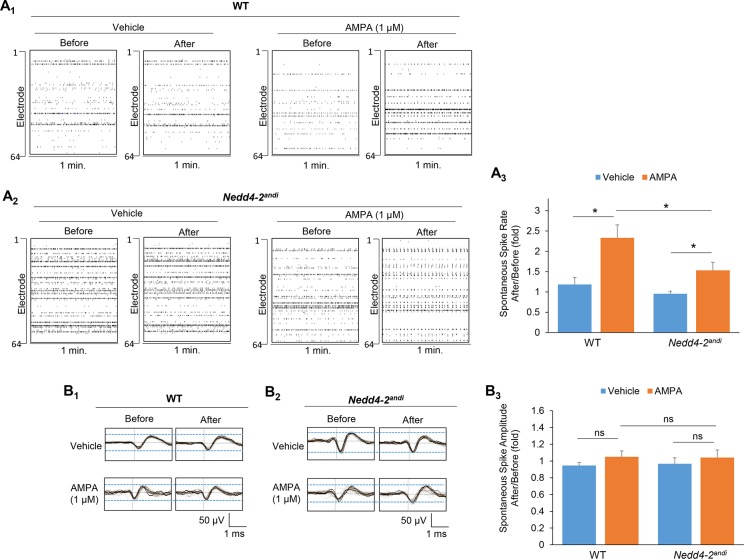
Acute AMPAR activation triggers stronger elevation of spontaneous neuronal activity in WT than *Nedd4-2*^*andi*^ cortical neuron cultures. (**A**) Representative raster plots and quantification of spontaneous spikes from 1-min recording (right) of (**A**_**1**_) WT or (**A**_**2**_) *Nedd4-2*^*andi*^ cortical neuron cultures before and after vehicle (ddH_2_O) or AMPA (1 μM) treatment for 15 min. (Vehicle: n = 6 and 9 for WT and *Nedd4-2*^*andi*^; AMPA: n = 7 and 11 for WT and *Nedd4-2*^*andi*^) (**B**) Representative average traces of 1-min recording and quantification of average spontaneous spike amplitude of (**B**_**1**_) WT or (**B**_**2**_) *Nedd4-2*^*andi*^ cortical neuron cultures before and after vehicle (ddH_2_O) or AMPA (1 μM) treatment for 15 min (Vehicle: n = 6 and 9 for WT and *Nedd4-2*^*andi*^; AMPA: n = 7 and 11 for WT and *Nedd4-2*^*andi*^). The black lines represent the average of all the spikes within representative 1-min recordings. Data are analyzed by a 2-way ANOVA with post-hoc Tukey test and represented as mean ± SEM. The comparison between treatments or genotypes is described with *p<0.05, ns: non-significant. Significant interaction between treatment and genotype was detected in spontaneous spike rate (A_3_; p<0.05) but not amplitude (B_3_; p>0.05).

We previously identified the GluA1 subunit of AMPAR as a substrate of Nedd4-2 [15]. We therefore asked whether AMPAR mediates spontaneous neuronal activity and whether it is responsible for the hyperactivity observed in *Nedd4-2*^*andi*^ cultures. An AMPAR agonist, AMPA (1 μM), was applied to determine how spontaneous neuronal activity was affected when AMPAR was activated. MEA recordings from WT cultures before and after AMPA treatment for 15 min indicated elevated spontaneous spike frequency (Fig 2A_1_ and 2A_3_) suggesting that spontaneous neuronal activity can be modulated by AMPARs. The same treatment produced significant, but smaller, effects on *Nedd4-2*^*andi*^ cultures (Fig 2A_2_ and 2A_3_; Significant interaction between treatment and genotype was detected, p<0.05.). The average of spontaneous spike amplitude (Fig 2B) and electrode burst activity did not differ after AMPA treatment for either genotype (S2A Fig). These data suggest that *Nedd4-2* contributes to the elevation of spontaneous neuronal activity, particularly spontaneous spike frequency, when the AMPAR is activated.

**Fig 3:**
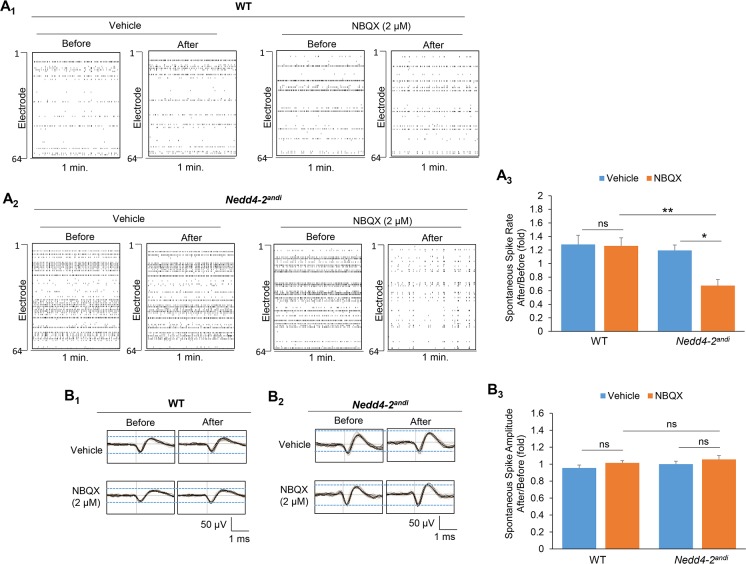
*Nedd4-2*^*andi*^ cortical neuron cultures are more sensitive to acute AMPAR blockade than WT cultures in reduction of spontaneous neuronal activity. (**A**) Representative raster plots and quantification of spontaneous spikes from 1-min recording (right) of (**A**_**1**_) WT or (**A**_**2**_) *Nedd4-2*^*andi*^ cortical neuron cultures before and after vehicle (DMSO) or NBQX (2 μM) treatment for 15 min. (Vehicle: n = 9 and 5 for WT and *Nedd4-2*^*andi*^; NBQX: n = 8 and 5 for WT and *Nedd4-2*^*andi*^) (**B**) Representative average traces of 1-min recording and quantification of average spontaneous spike amplitude of (**B**_**1**_) WT or (**B**_**2**_) *Nedd4-2*^*andi*^ cortical neuron cultures before and after vehicle (DMSO) or NBQX (2 μM) treatment for 15 min (Vehicle: n = 9 and 5 for WT and *Nedd4-2*^*andi*^; NBQX: n = 8 and 5 for WT and *Nedd4-2*^*andi*^). The black lines represent the average of all the spikes within representative 1-min recordings. Data are analyzed by a 2-way ANOVA with post-hoc Tukey test and represented as mean ± SEM. The comparison between treatments or genotypes is described with **p<0.01, ns: non-significant. Significant interaction between treatment and genotype was detected in spontaneous spike rate (A_3_; p<0.05) but not amplitude (B_3_; p>0.05).

We then asked whether *Nedd4-2*^*andi*^ cultures respond differently when AMPARs are pharmacologically inhibited. We employed a specific AMPAR antagonist, NBQX (2 μM), and again recorded the spontaneous neuronal activity before and after a 15-min treatment. As shown in Fig 3A, NBQX slightly, but not significantly, reduced spontaneous spike frequency in WT cultures, while NBQX significantly reduced spike frequency in *Nedd4-2*^*andi*^ cultures (Significant interaction between treatment and genotype was detected, p<0.05.). Again, average spontaneous spike amplitude did not differ between either treatments or genotypes (Fig 3B). Interestingly, we observed elevated burst activity without changes in interburst interval (IBI) after NBQX treatment (S2B Fig), which was also observed in another study [35]. Although we suspect the lack of changes in IBI is potentially because NBQX was applied acutely, we did not pursue further experiments with prolonged treatments since our data suggest that NBQX-induced burst activity is independent of *Nedd4-2* (S2B_1_ and S2B_2_ Fig).

We previously showed elevated synchrony of spontaneous neuronal activity in *Nedd4-2*^*andi*^ cultures [16]. Elevation of synchronized activity indicates potentially elevated network activity. To determine whether AMPAR is involved in this phenomenon, we analyzed the synchrony index in WT and *Nedd4-2*^*andi*^ cultures treated with either AMPA or NBQX. As shown, although AMPA treatment elicits some effect toward elevation of synchrony, no difference was observed between genotypes. NBQX, on the other hand, produces no effect on synchrony in either WT or *Nedd4-2*^*andi*^ cultures (S3 Fig). These results suggest that, although prolonged stimulation of AMPAR might further elevate the synchrony of neuronal activity, the effect is unlikely to be Nedd4-2-dependent. Furthermore, AMPAR is also unlikely to be responsible for basally elevated synchrony when Nedd4-2 is compromised [16] since NBQX exerts no effect. Therefore, whether and how Nedd4-2 mediates synchrony of spontaneous neuronal activity or other network activity, such as network spikes and bursts, through other substrates would be an important future direction.

In summary, we showed that *Nedd4-2*^*andi*^ cultures were less sensitive to AMPAR activation but very sensitive to AMPAR blockade with regard to spontaneous spike frequency. These results suggest that altered GluA1/AMPAR signaling in *Nedd4-2*^*andi*^ mice contributes to basally elevate spontaneous neuronal activity.

### Nedd4-2-associated seizures can be normalized when GluA1 is genetically reduced

**Fig 4:**
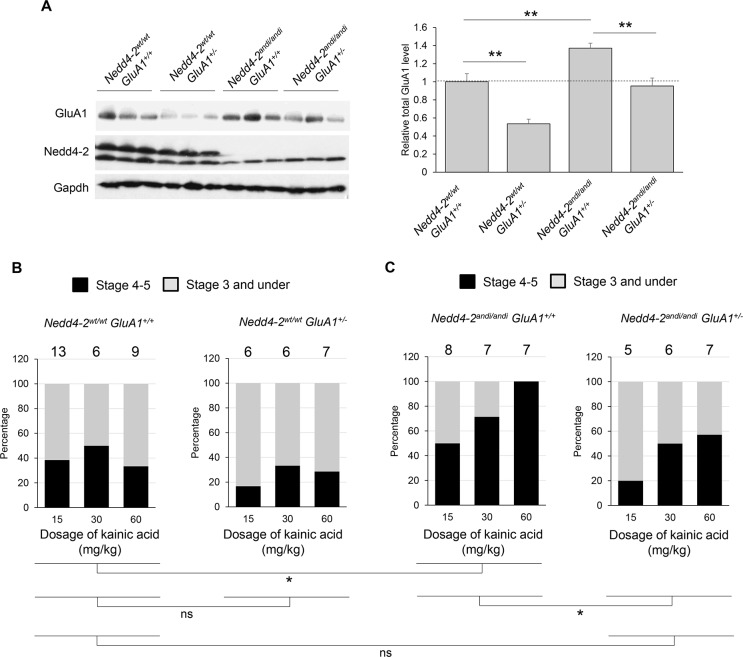
Genetically reducing GluA1 normalizes elevated seizure susceptibility in *Nedd4-2*^*andi*^ mice. (**A**) Western blots of GluA1, Nedd4-2, and Gapdh from 3 representative mice of each of the 4 genotypes: *Nedd4-2*^*wt/wt*^ *GluA1*^*+/+*^, *Nedd4-2*^*wt/wt*^ *GluA1*^*+/-*^, *Nedd4-2*^*andi/andi*^ *GluA1*^*+/+*^, and *Nedd4-2*^*andi/andi*^ *GluA1*^*+/-*^. Quantification of GluA1 is shown on the right (Six mice of each genotype were obtained for quantification. ANOVA with post-hoc Tukey test, **p < 0.01). (**B**) Four-week old mice with each of the 4 genotypes mentioned in (**A**) were intraperitoneally injected with kainic acid (15, 30, or 60 mg/kg). The number of mice used in each condition is shown on the top of each bar. Mice showing stage 4 and/or 5 seizure activity are counted as 1 while mice showing stage 3 seizure or under are counted as 0. The statistical results after a 2-way ANOVA with post-hoc Tukey test are as below: *Nedd4-2*^*wt/wt*^ *GluA1*^*+/+*^ vs *Nedd4-2*^*andi/andi*^ *GluA1*^*+/+*^, *p < 0.05; *Nedd4-2*^*andi/andi*^ *GluA1*^*+/+*^ vs *Nedd4-2*^*andi/andi*^ *GluA1*^*+/-*^, *p < 0.05; *Nedd4-2*^*wt/wt*^ *GluA1*^*+/+*^ vs *Nedd4-2*^*wt/wt*^ *GluA1*^*+/-*^, *p* > 0.05; *Nedd4-2*^*wt/wt*^ *GluA1*^*+/+*^ vs *Nedd4-2*^*andi/andi*^ *GluA1*^*+/-*^, *p > 0.05.

We have previously demonstrated that *Nedd4-2*^*andi*^ mice exhibit greater seizure susceptibility induced by systematic administration of kainic acid, a potent agonist for kainate-class ionotropic glutamate receptors that is widely used to induce seizures in animal models [16, 36]. Greater sensitivity to AMPAR blockade in reducing spontaneous neuronal activity, as seen in Fig 3, led to our hypothesis that elevated GluA1 level contributes to elevated seizure susceptibility in *Nedd4-2*^*andi*^ mice. To test this hypothesis, WT or *Nedd4-2*^*andi*^ mice were crossed with GluA1 knockout mice to obtain the following four genotypes: 1) *Nedd4-2*^*wt/wt*^ *GluA1*^*+/+;*^, 2) *Nedd4-2*^*wt/wt*^ *GluA1*^*+/-*^, 3) *Nedd4-2*^*andi/andi*^ *GluA1*^*+/+*^, and 4) *Nedd4-2*^*andi/andi*^ *GluA1*^*+/-*^. As shown in Fig 4A, GluA1 levels in *Nedd4-2*^*wt/wt*^ *GluA1*^*+/-*^ and *Nedd4-2*^*andi/andi*^ *GluA1*^*+/-*^ mice were reduced by 31% and 46%, respectively, when compared to their control littermates (*Nedd4-2*^*wt/wt*^ *GluA1*^*+/+*^ and *Nedd4-2*^*andi/andi*^ *GluA1*^*+/+*^, respectively). Most importantly, the GluA1 level in *Nedd4-2*^*andi/andi*^ *GluA1*^*+/-*^ mice was similar to *Nedd4-2*^*wt/wt*^ *GluA1*^*+/+*^ mice (Fig 4A). We then determined seizure susceptibility in these mice by intraperitoneal injections of kainic acid. Four-week-old mice were injected with kainic acid (15, 30, or 60 mg/kg) as done in our previous study [16]. Behavioral seizures were monitored and scored during a 1-hr observation period. As shown, *Nedd4-2*^*andi*^ mice (*Nedd4-2*^*andi/andi*^ *GluA1*^*+/+*^; Fig 4C, left panel) had enhanced seizure response in comparison to WT mice (*Nedd4-2*^*wt/wt*^ *GluA1*^*+/+*^; Fig 4B, left panel). Reducing GluA1 levels in *Nedd4-2*^*andi*^ mice (*Nedd4-2*^*andi/andi*^ *GluA1*^*+/-*^; Fig 4C, right panel) significantly reduced seizure response in comparison to control *Nedd4-2*^*andi*^ mice (*Nedd4-2*^*andi/andi*^ *GluA1*^*+/+*^; Fig 4C, left panel). Furthermore, reducing GluA1 level in *Nedd4-2*^*andi*^ mice produced a seizure response similar to WT mice (*Nedd4-2*^*wt/wt*^ *GluA1*^*+/+*^; Fig 4B, left panel). A slight, but not significant, reduction of seizure response was also observed when GluA1 level was reduced in WT mice (*Nedd4-2*^*wt/wt*^ *GluA1*^*+/-*^; Fig 4B, right panel). In conclusion, our results indicate that genetically reducing GluA1 level is able to correct elevated seizure susceptibility caused by insufficient function of Nedd4-2 in *Nedd4-2*^*andi*^ mice.

### Nedd4-2 ubiquitinates GluA1 at lysine-868 and mediates its surface expression

**Fig 5:**
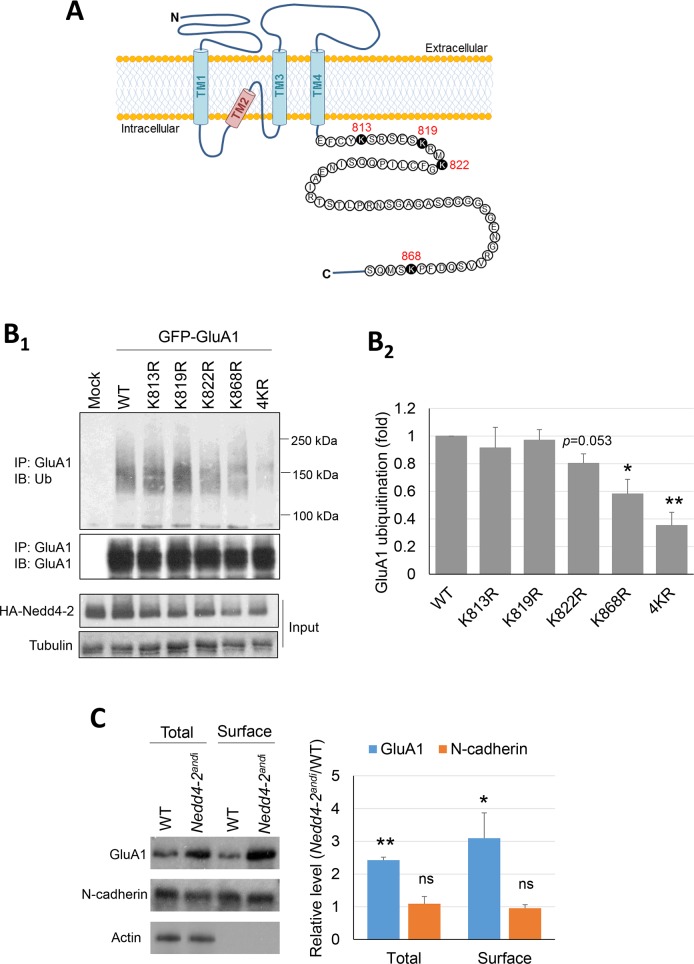
Nedd4-2 mediates surface expression of GluA1. (**A**) Illustration of GluA1 on the cell membrane and the 4 lysine residues potentially ubiquitinated by Nedd4-2 at the C-terminus of GluA1. (**B**_**1**_) Western blots of Ubiquitin (Ub) or GluA1 after immunoprecipitation using anti-GluA1 antibody from HEK cells transfected with WT HA-Nedd4-2 along with WT GluA1 or mutant GluA1s (K813R, K819R, K822R, K868R and 4KR) for 48 hours. Quantification of ubiquitinated GluA1 by the area of smear from 100–250 kDa is shown on the right (**B**_**2**_) (n = 4, one-way ANOVA with post-hoc Tukey test). (**C**) Western blots of GluA1, N-cadherin, and Actin from WT or *Nedd4-2*^*andi*^ cortical neuron cultures. Proteins from total lysate or after surface biotinylation were as indicated (n = 3, one-sample *t*-test was performed after normalization to WT groups). For all experiments, data are represented as mean ± SEM with *p<0.05, **p<0.01.

Our data suggest that impaired GluA1 ubiquitination may be responsible for Nedd4-2-mediated seizure and/or epilepsies. To test this hypothesis, we sought to characterize the functional consequence of GluA1 ubiquitination by Nedd4-2. We first attempted to map the Nedd4-2-ubiquitinated residues of GluA1 as ubiquitination at different residues may affect the function of GluA1 differently [20, 21]. We employed human embryonic kidney (HEK) cells because they do not express a detectable level of GluA1 or Nedd4-2 endogenously (S4 Fig). There are four lysine residues (K813, K819, K822, and K868) located on the carboxyl-terminal, intracellular domain of GluA1 (Fig 5A) [21–23]. As reported previously, mutating all four residues completely abolishes GluA1 ubiquitination; this rules out other lysine residues as targets [21]. Accordingly, WT Nedd4-2 was then co-transfected with WT GluA1 or mutant GluA1s in which each lysine was replaced by arginine (R) at each individual site (K813R, K819R, K822R and K868R) or all four lysine residues together were mutated to R (4KR). As shown in Fig 5B, the GluA1 with either K868R or all four lysine residues mutated to arginine (4KR) showed significantly reduced ubiquitination when co-expressed with Nedd4-2. A trend toward reduced ubiquitination is also observed when GluA1 carries K822R, suggesting a potential alternative residue for Nedd4-2-mediated ubiquitination. In summary, these results suggest that K868 is the most critical residue ubiquitinated by Nedd4-2.

GluA1 ubiquitination at K868 has been shown to affect its surface expression [22, 23]. To determine whether Nedd4-2 mediates surface expression of GluA1, we labeled surface proteins with biotin in WT or *Nedd4-2*^*andi*^ cortical neuron cultures followed by purification of biotinylated proteins with streptavidin beads. As shown in Fig 5C, *Nedd4-2*^*andi*^ cultures indeed exhibited elevated surface GluA1 when compared with WT cultures. N-cadherin serves as a control and did not differ between WT or *Nedd4-2*^*andi*^ cultures. These results confirm the role of Nedd4-2 in limiting GluA1 surface expression. Because elevated surface GluA1 level has been linked to enhanced seizure susceptibility [37, 38], our findings further support our hypothesis that altered GluA1 ubiquitination contributes to Nedd4-2-associated seizures and/or epilepsies.

### Three epilepsy-associated missense mutations reduce Nedd4-2-mediated GluA1 ubiquitination

**Fig 6:**
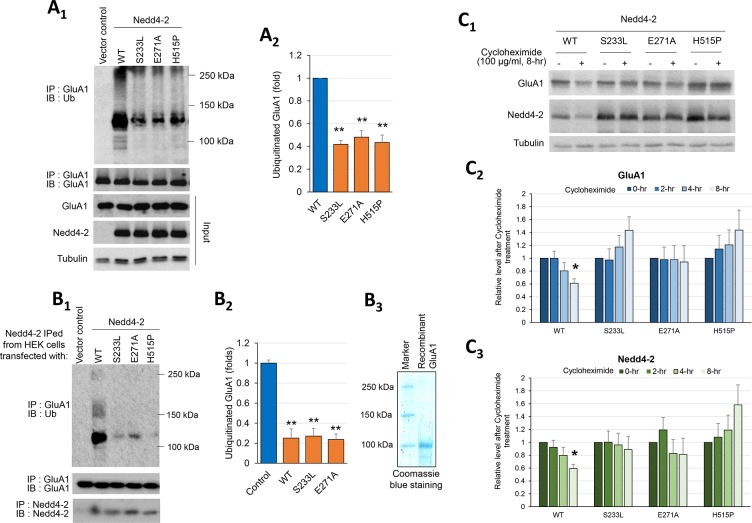
Three epilepsy-associated missense mutations of Nedd4-2 reduce GluA1 ubiquitination. (**A**_**1**_) Western blots of Ubiquitin (Ub) or GluA1 after immunoprecipitation using anti-GluA1 antibody from HEK cells transfected with GluA1 along with HA-tagged WT or mutant Nedd4-2 for 48 hours. Quantification of ubiquitinated GluA1 by the entire area of smear from 100–250 kDa is shown on the right (**A**_**2**_) (n = 4, one-way ANOVA with post-hoc Tukey test). (**B**_**1**_) Western blots of Ub or GluA1 after immunoprecipitation with anti-GluA1 antibody following *in vitro* ubiquitination with recombinant GluA1. HA-tagged WT or mutant Nedd4-2s used for *in vitro* ubiquitination were obtained from HEK cells transfected with one of the Nedd4-2s followed by immunoprecipitation with anti-Nedd4-2 antibody. Quantification of ubiquitinated GluA1 by the entire area of smear (**B**_**2**_) and Coomassie blue staining showing the purity of recombinant GluA1 (**B**_**3**_) are shown (n = 4, one-way ANOVA with post-hoc Tukey test). (**C**_**1**_) Western blots of GluA1 and Nedd4-2 after cycloheximide treatment over an 8-hr time course. HEK cells were transfected with GluA1 and HA-tagged WT or mutant Nedd4-2s for 48 hours. Cells were then treated with cycloheximide (100 μg/ml) to inhibit protein translation and follow protein degradation. Representative western blots after 0- and 8-hr cycloheximide treatment (**C**_**1**_) and time courses of GluA1 (**C**_**2**_) and Nedd4-2 (**C**_**3**_) levels after cycloheximide treatment are shown. Analyses were performed by comparing GluA1 or Nedd4-2 level at each time point between cultures receiving different Nedd4-2s (n = 4, one-way ANOVA with post-hoc Tukey test). For all experiments, data are represented as mean ± SEM with *p<0.05, **p<0.01.

There are three epilepsy-associated missense mutations of Nedd4-2 (S233L, E271A, and H515P) identified in patients with epilepsies [4, 5]. Based on our findings, we aimed to test the hypothesis that one or more of these mutations could disrupt GluA1 ubiquitination. To avoid potential inference from other neuronal E3 ligases for GluA1 [39, 40], we applied reconstitutive systems to determine GluA1 ubiquitination using either HEK cells or *in vitro* ubiquitination. When using HEK cells co-transfected with GluA1 and Nedd4-2, we found that GluA1 is less ubiquitinated when co-expressed with any of the Nedd4-2 mutants in comparison to WT Nedd4-2 (Fig 6A and S5 Fig). When *in vitro* ubiquitination was performed using recombinant full-length GluA1 with WT or any of the expressed Nedd4-2 mutants (partially purified from HEK cells), we also found that in comparison to WT Nedd4-2, all three mutant Nedd4-2s exhibited reduced ability to ubiquitinate GluA1 *in vitro* (Fig 6B).

Previously we showed that Nedd4-2-mediated-GluA1 ubiquitination leads to degradation of GluA1 [15]. To determine whether mutant Nedd4-2s fail to degrade GluA1, HEK cells were co-transfected with GluA1 and WT or mutant Nedd4-2. Using cycloheximide (100 μg/ml) to inhibit protein translation and follow protein degradation, significant GluA1 down-regulation is only observed when co-expressed with WT Nedd4-2 but not with any of the mutant Nedd4-2s (Fig 6C). Slightly enhanced levels of GluA1 or Nedd4-2 after cycloheximide treatment were observed in some groups after normalization with the internal control Tubulin. This is due to a lower turnover rate of GluA1 or Nedd4-2 than that of Tubulin when cells were transfected with mutant Nedd4-2s. To strengthen the idea that GluA1 degradation is altered when co-expressed with mutant Nedd4-2s, HEK cells co-transfected with GluA1 and WT or mutant Nedd4-2 were treated with proteasome inhibitor MG132 (10 μM) (S6 Fig). GluA1 significantly accumulates when co-expressed with WT Nedd4-2, but not with any of the mutant Nedd4-2s, after MG132 treatment. Altogether, our data suggest that all three missense mutations disrupt Nedd4-2-mediated GluA1 ubiquitination and degradation.

### Three epilepsy-associated missense mutations of Nedd4-2 disrupt 14-3-3-facilitated GluA1 ubiquitination

**Fig 7:**
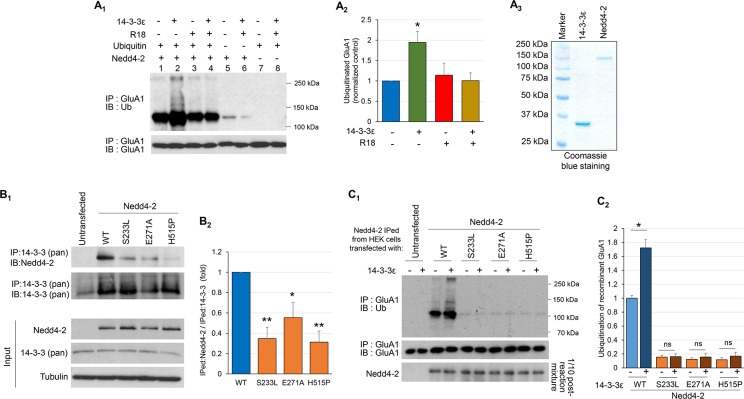
Three epilepsy-associated missense mutations of Nedd4-2 disrupt 14-3-3-facilitated GluA1 ubiquitination. (**A**_**1**_) Western blots of Ub or GluA1 after immunoprecipitation with anti-GluA1 antibody following *in vitro* ubiquitination with recombinant GluA1 and Nedd4-2, and in the presence or absence of recombinant 14-3-3ε, 14-3-3 inhibitor R18, or Ubiquitin as labeled. Quantification of lanes 1–4 by the entire area of smear from 100–250 kDa (**A**_**2**_) and Coomassie blue staining showing the purity of recombinant Nedd4-2 and 14-3-3ε (**A**_**3**_) (n = 4, 2-way ANOVA with post-hoc Tukey test). (**B**_**1**_) Western blots of Nedd4-2 or 14-3-3 after a co-immunoprecipitation using anti-14-3-3 antibody with the lysate of HEK cells transfected with HA-tagged WT or mutant Nedd4-2s for 48 hours. Input of transfected Nedd4-2s, endogenous 14-3-3, and Tubulin are shown on the bottom. Quantification of immunoprecipitated Nedd4-2 (**B**_**2**_) is shown on the right (n = 4, one-way ANOVA with post-hoc Tukey test). (**C**_**1**_) Western blots of Ub or GluA1 after immunoprecipitation with anti-GluA1 antibody following *in vitro* ubiquitination with recombinant GluA1 in the presence or absence of recombinant 14-3-3ε. HA-tagged WT or mutant Nedd4-2s used for *in vitro* ubiquitination were obtained from HEK cells transfected with one of the Nedd4-2s followed by immunoprecipitation with anti-Nedd4-2 antibody. Right before the washing, 1/10 of reaction mixture was obtained and used as input control shown on the bottom. (**C**_**2**_) The intensity of ubiquitinated GluA1 by the entire area of smear from 100–250 kDa is normalized to the WT group in the absence of 14-3-3ε (lane 3 on the representative blot C_1_). The difference in each group with or without the addition of 14-3-3ε was analyzed by Student’s *t*-test. For all experiments, data are represented as mean ± SEM with *p<0.05, **p<0.01.

When expressing WT or mutant Nedd4-2s in HEK cells, it was observed that the mutant Nedd4-2s exhibited increased basal levels and reduced degradation when compared with WT Nedd4-2 (Fig 6C_3_ and S6 Fig), suggesting enhanced stability. Because Nedd4-2 can self-ubiquitinate, the reduced down-regulation of both GluA1 and Nedd4-2 suggests that these missense mutations most likely affect the general ubiquitination process mediated by Nedd4-2 [41, 42]. Furthermore, all three mutations are located on or near one of the three protein-protein interaction domains (WW domain) in Nedd4-2 (S7 Fig), suggesting potentially altered interaction with its interacting proteins. To test this possibility, we studied the adaptor protein 14-3-3, which directly interacts with Nedd4-2 and has been shown to mediate Nedd4-2’s substrate recognition [43–45]. We first aimed to determine whether 14-3-3 mediates Nedd4-2-mediated GluA1 ubiquitination. *In vitro* ubiquitination using recombinant GluA1 and Nedd4-2 yielded some GluA1 ubiquitination (Fig 7A_1_, lane 1). Remarkably, in the presence of recombinant 14-3-3ε, one of the 14-3-3 isoforms known to interact with Nedd4-2 [46], GluA1 ubiquitination was significantly enhanced (Fig 7A_1_, lane 2). To validate the role of 14-3-3, a peptide-based general 14-3-3 inhibitor, R18 trifluoroacetate (R18; 0.025 mg/ml), which is known to disrupt the interaction between 14-3-3 and its binding partners, was used [47–49]. As predicted, R18 reduced GluA1 ubiquitination to a level similar to Nedd4-2 alone (Fig 7A_1_, lane 4). For controls, the same reactions in the absence of either Nedd4-2 or ubiquitin showed nearly undetectable GluA1 ubiquitination (Fig 7A_1_, lanes 5–8). These data suggest that, while Nedd4-2 is capable of ubiquitinating GluA1 in the absence of 14-3-3, 14-3-3 significantly facilitates this ubiquitination.

We then asked whether reduced GluA1 ubiquitination by epilepsy-associated missense mutations of Nedd4-2 occurred through altered interactions with 14-3-3. WT or mutant Nedd4-2 was transfected into HEK cells. Co-immunoprecipitation showed that all three mutants have reduced interactions with endogenous 14-3-3 in HEK cells (Fig 7B). Similar results were obtained when using recombinant 14-3-3ε to immunoprecipitate WT or mutant Nedd4-2 expressed in HEK cells (S8 Fig). To determine whether the Nedd4-2 mutants fail to respond to 14-3-3 when ubiquitinating GluA1, *in vitro* ubiquitination using recombinant GluA1 and WT or mutant Nedd4-2 partially purified from HEK cells was performed. While WT Nedd4-2 strongly ubiquitinated GluA1 and responded to additional 14-3-3ε with further GluA1 ubiquitination, all of the mutant Nedd4-2s failed to do so (Fig 7C). Because the level of 14-3-3 does not seem to be regulated by Nedd4-2 (S9 Fig), our results suggest that the epilepsy-associated missense mutations of Nedd4-2 disrupt GluA1 ubiquitination, at least partially through reduced interaction with 14-3-3.

### Three epilepsy-associated missense mutations of Nedd4-2 fail to regulate surface GluA1 and spontaneous neuronal activity

**Fig 8:**
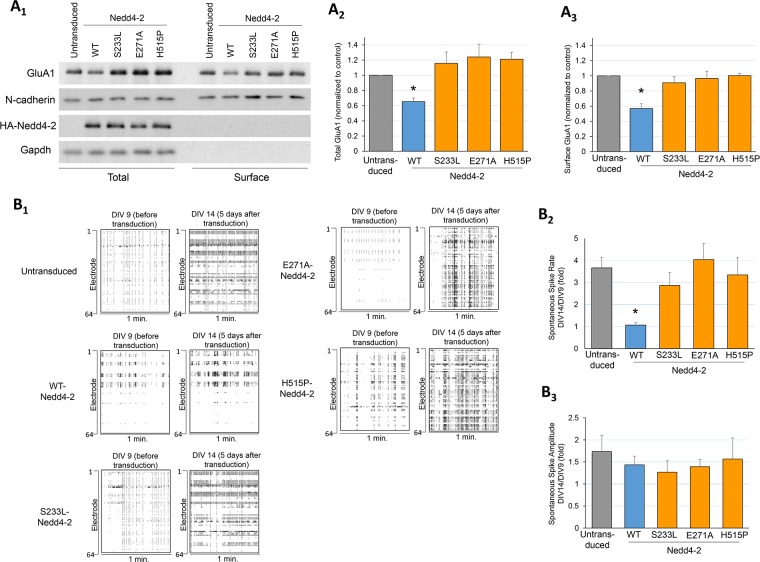
Three epilepsy-associated missense mutations of Nedd4-2 fail to mediate surface GluA1 level and spontaneous neuronal activity. (**A**_**1**_) Western blots of GluA1, N-cadherin, HA-Nedd4-2, and Actin from *Nedd4-2*^*andi*^ cultures lentivirally transduced with or without WT or mutant Nedd4-2s for 5 days starting at DIV 9. Proteins from total lysate or after surface biotinylation were as indicated. Quantification of (**A**_**2**_) total and (**A**_**3**_) surface GluA1 are on the right (n = 4, one-way ANOVA with post-hoc Tukey test). (**B**_**1**_) Representative raster plots, (**B**_**2**_) quantification of spontaneous spike rate from 1-min recording and (**B**_**3**_) quantification of average spontaneous spike amplitude of *Nedd4-2*^*andi*^ cortical neuron cultures lentivirally transduced with or without WT or mutant Nedd4-2s for 5 days starting at DIV 9 (n = 5–7, one-way ANOVA with post-hoc Tukey test). Data are represented as mean ± SEM with *p<0.05, ns: non-significant.

Because the epilepsy-associated missense mutations of Nedd4-2 disrupt GluA1 ubiquitination and degradation, we hypothesize that these mutations fail to mediate surface GluA1 and spontaneous neuronal activity. To this end, we performed surface protein biotinylation to obtain and measure surface GluA1 from WT cortical neuron cultures lentivirally transduced with WT or mutant Nedd4-2 for 5 days. Surprisingly, no significant effect was observed (S10 Fig). We suspect that the level of Nedd4-2 during early development might reach a threshold in WT cultures, and therefore expression of additional Nedd4-2 fails to elicit significant effects. Furthermore, because mutant Nedd4-2s exhibit significantly reduced affinity toward interacting with 14-3-3 and therefore GluA1 (Figs 6 and 7), they might not be dominant-negative, at least in the context of GluA1 ubiquitination. The endogenous Nedd4-2 in WT cultures potentially dominates even in the presence of mutant Nedd4-2s. Therefore, to determine the effects of mutant Nedd4-2s without the interference from endogenous Nedd4-2, we repeated this experiment in *Nedd4-2*^*andi*^ cortical neuron cultures. Using *Nedd4-2*^*andi*^ cultures possesses the advantage of studying the behavior of mutant Nedd4-2s while minimizing the concern of overexpression. As shown in Fig 8A, WT Nedd4-2 significantly reduced total and surface GluA1 in *Nedd4-2*^*andi*^ cultures while all three mutant Nedd4-2s showed no effect. When expressing WT Nedd4-2 or any of the mutant Nedd4-2s in *Nedd4-2*^*andi*^ cortical neuron cultures for 5 days, we found that the cultures transduced with WT Nedd4-2 showed significantly lower spontaneous spike frequency when compared with untransduced cultures or cultures transduced with any of the mutant Nedd4-2s (Fig 8B_1_ and 8B_2_). The average spontaneous spike amplitude did not differ between transduced and untransduced cultures. Altogether, our data showed that the three epilepsy-associated missense mutations of Nedd4-2 disrupt the ability to regulate surface GluA1 and spontaneous neuronal activity.

## Discussion

In this study, we present evidence to show that neuronal hyperactivity *in vitro* and increased seizure susceptibility *in vivo* associated with *Nedd4-2* dysfunction are modulated by altered GluA1 and AMPAR signaling. These findings are further supported by the data showing that three epilepsy-associated missense mutations of Nedd4-2 partially, but significantly, disrupted GluA1 ubiquitination through reduced interaction with the adaptor protein 14-3-3. All mutant Nedd4-2s retain partial function toward ubiquitinating GluA1, reflecting the fact that the mutations were located on or near protein-protein interaction domains but not the lipid-binding or catalytic HECT (Homologous to the E6-AP Carboxyl Terminus) domain. Nevertheless, this is the first report that demonstrates a mechanism to explain *Nedd4-2*-dependent epilepsy in patients. Although the mutations of Nedd4-2 increase its stability, which is different from the *in vivo* knockdown mouse model we used (*Nedd4-2*^*andi*^ mice), we showed that the reduction of Nedd4-2 and the mutations each reduce the ability of Nedd4-2 to ubiquitinate GluA1. Furthermore, because our data showed that genetic reduction of GluA1 normalized the seizure response in *Nedd4-2*^*andi*^ mice, it suggests that inhibition of AMPARs might be a suitable treatment plan for *Nedd4-2*-associated epilepsy. One of the antagonists of AMPAR, Perampanel, has been approved to clinically reduce partial-onset seizures with or without secondary generalized seizures in epileptic patients [50–52]. Such medication might therefore be specifically useful for epilepsy patients who carry mutations in *Nedd4-2*. A future study on Perampanel will be very important to determine whether and to what the extent *Nedd4-2*-associated seizures and/or epilepsy can be ameliorated.

We used *Nedd4-2*^*andi*^ mice, in which the long form of *Nedd4-2* is disrupted, to study *Nedd4-2*. Because *Nedd4-2* knockout mice exhibit perinatal lethality [34], *Nedd4-2*^*andi*^ mice serve as an ideal model to study *Nedd4-2 in vivo*. However, another question is thus raised regarding the differential contribution of long versus short form of Nedd4-2 to the regulation of spontaneous neuronal activity and brain circuit excitability. The short form of Nedd4-2, which lacks an N-terminal C2 domain (S7 Fig), has also been identified in humans [53, 54]. Indirect evidence has suggested that the C2 domain mediates membrane-targeting of Nedd4-2 [8]. The C2-containing (long) and C2-lacking (short) isoforms therefore target different intracellular locations and substrate pools [8]. Currently, it is unclear whether the short form of Nedd4-2 exhibits similar affinity toward binding to and ubiquitinating GluA1. If it does, the question arises as to whether epilepsy-associated mutations affect the function of short form Nedd4-2 in a similar manner as to the long form of Nedd4-2. If it does not, the second question is whether the short form serves as a dominant-negative Nedd4-2 to sequester interacting or signaling molecules to affect the functions of the long form of Nedd4-2. Because single-nucleotide polymorphisms (SNPs) in human Nedd4-2 lead to differential expression of these isoforms, examining the functional differences of these isoforms may increase our understanding of neuronal plasticity and associated seizure susceptibility in different populations [53, 54].

In the adult brain, the AMPAR has been shown to mediate the majority of excitatory synaptic transmission with GluA1 being one of the major subunits [55]. Activity-mediated changes in the numbers and properties of GluA1/AMPAR are essential for excitatory synapse development and synaptic plasticity. Ubiquitination of GluA1 has been linked to AMPAR surface expression and trafficking, which subsequently may affect many synaptic plasticity mechanisms, such as homeostatic synaptic scaling and synaptic depression [21–23, 56, 57]. We previously demonstrated that Nedd4-2 mediates GluA1 ubiquitination upon chronic neuronal activity stimulation, suggesting a potential role of Nedd4-2 in homeostatic synaptic downscaling [15]. Whether Nedd4-2 participates in other synaptic plasticity mechanisms is unknown. One speculation would be that because Nedd4-2 functions to limit the amount of surface GluA1 as seen in Fig 5C, neuronal activity that mediates depression or elimination of excitatory synapses might induce Nedd4-2-mediated GluA1 ubiquitination. We recently found that the expression of Nedd4-2 is modulated by another ubiquitin E3 ligase murine double minute-2 (Mdm2) and its downstream effector tumor suppressor p53 [15]. Mdm2 is known to be crucial for activity-dependent synapse elimination [58], which is crucial for brain circuit development and maturation. Activation of Mdm2-p53 signaling and Nedd4-2 expression might therefore contribute to elimination of excitatory synapses. Activation of Mdm2-p53 signaling and Nedd4-2 expression might therefore contribute to elimination of excitatory synapses. Further studies are required to delineate the broader effects of Nedd4-2.

In addition to GluA1, other neuronal substrates of Nedd4-2 potentially involved in neuronal activity regulation are voltage-gated sodium channels Na_v_1.6 and voltage-gated potassium channels K_v_7.3/KCNQ3 [9–12]. These two substrates are both crucial to modulating action potential firing and intrinsic excitability. Although our data showed that GluA1 mediates Nedd4-2-associated neuronal hyperactivity and seizures in mice, it does not rule out the potential contributions of Na_v_1.6 and K_v_7.3/KCNQ3 in Nedd4-2-associated brain circuit excitability. Our data also suggest that presynaptic defects are potentially involved in the neuronal deficits associated with Nedd4-2 (Fig 1D). Multiple substrates of Nedd4 family members are known to mediate presynaptic vesicle release and activity, including α-synuclein [59–61] and tyrosine kinase A receptors [13, 14, 62]. Altered ubiquitination of these substrates when Nedd4-2’s function is compromised could contribute to aberrant synaptic transmission. The ubiquitination status, expression level, and subcellular distribution of Nedd4-2’s other substrates are pending further investigation to obtain the full picture of synaptic abnormality and excitability caused by pathogenic functions of mutant Nedd4-2s. As we described previously, future studies are expected to elucidate broader effects of Nedd4-2 and provide better understanding of this important, yet underdeveloped, molecule in the central nervous system.

## Materials and methods

### Ethics statement

All experiments using animal data followed the guidelines of Animal Care and Use provided by the Illinois Institutional Animal Care and Use Committee (IACUC) and the guidelines of the Euthanasia of Animals provided by the American Veterinary Medical Association (AVMA) to minimize animal suffering and the number of animals used. This study was performed under an approved IACUC animal protocol of University of Illinois at Urbana-Champaign (#14139 to N.-P. Tsai.)

### Animals and primary cortical neuron cultures

The *Nedd4-2*^*andi*^ mice, GluA1 knockout mice and WT control mice were obtained from The Jackson Laboratory. Primary cortical neuron cultures were made from p0-p1 mice as described previously [58] and maintained in NeuralQ basal medium (Sigma) supplemented with 1X B27 supplement (Invitrogen), 1X GlutaMax (final concentration at 2 mM; Invitrogen), and Cytosine β-D-arabinofuranoside (AraC, final concentration at 2 μM; Sigma). The medium was changed 50% on DIV 2 and every 3 days thereafter.

### Reagents and constructs

Dimethyl sulfoxide (DMSO) was from Fisher Scientific. AMPA was from Cayman Chemical and NBQX was from Alomone Labs. Recombinant GluA1 and 14-3-3ε were from Origene. Recombinant Nedd4-2 was from Abnova. R18 was from Sigma-Aldrich. Cycloheximide, poly-D-lysine and Protein A/G beads were from Santa Cruz Biotechnology. The antibodies used in this study were purchased from Santa Cruz Biotechnology (anti-α-Tubulin), Cell Signaling (anti-Nedd4-2, anti-pan-14-3-3, anti-N-cadherin and anti-Ubiquitin), Millipore (anti-GluA1), Abcam (anti-MAP2), Thermo Scientific (anti-HA) and GenScript Corporation (anti-Gapdh). The epilepsy-associated mutations were generated using site-directed mutagenesis reagent (Agilent) to introduce mutations into pCI-HA-Nedd4-2 [15]. The primers used are as below.

S233L: 5’-GGACGTGTCCTCGGAGTTGGACAATAACATCAGAC-3’,

5’-GTCTGATGTTATTGTCCAACTCCGAGGACACGTCC-3’;

E271A: 5’- GGGCGGGGATGTCCCCGCGCCTTGGGAGACCATTTC-3’,

5’- GAAATGGTCTCCCAAGGCGCGGGGACATCCCCGCCC-3’;

H515P: 5’- CGTTTGAAATTTCCAGTACCTATGCGGTCAAAGACATC-3’,

5’- GATGTCTTTGACCGCATAGGTACTGGAAATTTCAAACG-3’.

### Surface protein biotinylation

After washing cultured cells with PBS three times, 0.1 mg LLC NHS-LC-BIOTIN (from Apexbio Technology) was added to cultures for 30 min. at room temperature. At the end of the reaction, cultures were washed with PBS three times. The cell were harvested and lysed in an IP buffer (50 mM Tris, pH 7.4, 120 mM NaCl, 0.5% Nonidet P-40) followed by purification using Magnetic Streptavidin Beads (from Cell Signaling).

### Transfection, immunoprecipitation and western blotting

HEK cells were transfected using Lipofectamine 3000 (Invitrogen). Primary neuron cultures were transduced using lentivirus. WT and mutant Nedd4-2s were sub-cloned into Lenti-CMV-GFP-2A-Puro Vector (from Applied Biological Materials). Lentivirus was produced in HEK cells as described previously [58].

For immunoprecipitation (IP), cell lysates were obtained by sonicating pelleted cells in IP buffer. Eighty μg of total protein or protein mixtures after *in vitro* ubiquitination was incubated 2 hours at 4°C with 0.5 μg primary antibodies. Protein A/G agarose beads were added for another hour followed by washing with IP buffer three times. For western blotting, after SDS-PAGE, the gel was transferred onto a polyvinylidene fluoride membrane. After blocking with 1% Bovine Serum Albumin in TBST buffer (20 mM Tris pH 7.5, 150 mM NaCl, 0.1% Tween-20), the membrane was incubated with primary antibody overnight at 4°C, followed by three 10-min washings with TBST buffer. The membrane was then incubated with an HRP-conjugated secondary antibody (from Santa Cruz Biotechnology) for 1 hour at room temperature, followed by another three 10-min washings. Finally, the membrane was developed with an ECL Chemiluminescent Reagent [15]. All the western blot results were semi-quantitatively normalized to the control groups before statistical analysis.

### In vitro ubiquitination

HA-Ub (Boston Biochem), Ubiquitin Activating Enzyme (UBE1) (Boston Biochem) and UbcH5b/UBE2D2 (Boston Biochem) were obtained. Recombinant WT Nedd4-2 was obtained from Abcam. When HA-tagged WT or mutant Nedd4-2s were produced in HEK cells, 250 μg of total protein lysates were subjected to immunoprecipitation with anti-HA antibody to partially purify HA-tagged Nedd4-2s. Recombinant GluA1 (Origene) was used as substrate for *in vitro* ubiquitination with recombinant Nedd4-2 (Fig 7A) or Nedd4-2s obtained from transfected HEK cells (Figs 6B and 7C) following a protocol previously described [63]. Recombinant 14-3-3ε was obtained from Origene.

### MEA recording

Each MEA plate was coated with poly-D-lysine for 30 minutes and plated with 2x10^5^ cells counted using a hemocytometer. Recordings were done at DIV 13–14 (Figs 1, 2 and 3) or DIV 9 and DIV 14 (Fig 8) in the same culture medium using an Axion Muse 64-channel system in single well MEAs (M64-GL1-30Pt200, Axion Biosystems) inside a 5% CO_2_, 37°C incubator. Field potentials (voltage) at each electrode relative to the ground electrode were recorded with a sampling rate of 25 kHz. Right before a recording, if an electrode channel displays excessive “noise” (>10 μV) that channel is grounded for the entirety of the recording to avoid interference with other channels [64]. After 30 min of baseline recording, the MEA was treated with the drugs specified and recorded for another 30 min. Due to changes in network activity caused by physical movement of the MEA, only the last 15 min of each recording were used in data analyses. AxIS software (Axion Biosystems) was used for the extraction of spikes (i.e. action potentials) from the raw electrical signal obtained from the Axion Muse system. After filtering, a threshold of ±7 standard deviations was independently set for each channel; activity exceeding this threshold was counted as a spike. Only MEAs with more than 2,000 spikes during the last 15 minutes of recording were included for data analysis [16, 65]. The total spikes obtained from each MEA culture was normalized to the number of electrodes, as described in a previous study [66].

The settings for burst detection in each electrode were a minimum of 5 spikes with a maximum inter-spike interval of 0.1 sec as described previously [16]. The burst duration, number of spikes per burst, and interburst interval were analyzed by AxIS software. Synchrony index was also computed through AxIS software, based on a published algorithm [67] as we conducted previously [16], by taking the cross-correlation between any two spike trains, removing the portions of the cross-correlogram that are contributed by the auto-correlations of each spike train, and reducing the distribution to a single metric.

To ensure consistency when acquiring MEA data, all the experiment procedures, including the animal dissection, cell counting and plating, medium changing, and recordings are conducted by the same individual in each experiment. Throughout culture maturation and before recording, each MEA is visually inspected under the microscope and any MEA with poor growth or a patchy network is excluded. Recordings of each experiment were alternate between genotypes. For all before and after drug treatment comparisons, to minimize the variability between cultures, the recording from each MEA culture after treatment was compared to the baseline recording from that same culture.

### Immunocytochemistry

Immunocytochemistry was done as previously described [68]. In brief, primary neurons grown on poly-D-lysine coated coverslips were fixed at DIV 14 with ice-cold buffer (4% paraformaldehyde and 5% sucrose in PBS). After washing and permeabilization with an additional incubation with 0.5% Triton X-100 in PBS for 5 min, an incubation with anti-MAP2 antibody was performed for 4 hours. After washing three times with PBS, fluorescence-conjugated secondary antibodies were applied to the cells at room temperature for 1 hour. After additionally washing the cells three times with PBS, the coverslips were mounted and observed on Zeiss LSM 700 Confocal Microscope.

### Seizure susceptibility

Male mice at age 4-weeks old were intraperitoneally injected with kainic acid, prepared in saline solution (Hannas Pharmaceutical), at doses of 15, 30, or 60 mg/kg. The total injection volume was kept close to 0.15 ml. After injection, mice were closely observed in real time for 1 hour. The intensity of seizure was assessed by Racine’s scoring system [69]. To clearly determine seizure susceptibility, only stage 4 (rearing) and stage 5 (rearing and falling) were considered positive for seizures, as previously performed [16, 70]. Mice showing stage 4 seizure and above are counted as 1 while mice showing stage 3 seizure or under are counted as 0 for analysis.

### Statistical analysis

ANOVA with post-hoc Tukey HSD (Honest Significant Differences) test was used for multiple comparisons between treatments or genotypes. Student’s *t*-test was used for analyzing spontaneous neuronal activity in Fig 1B and 1C, and mEPSC data in Fig 1D. One-sample *t*-test was used when experimental groups were normalized to control groups, such as western blotting in Fig 5C. Each “n” indicates an independent culture. Differences are considered significant at the level of *p* < 0.05.

## Supporting information

S1 Fig An example MEA culture. (**A**) A picture of a multi-electrode array (MEA) dish and (**B**) a representative image of cultured cortical neurons growing in a MEA dish at DIV 14. A selected area in (**B**_**1**_) is enlarged and shown in (**B**_**2**_). Images were acquired by an EPI-Fluorescence Trinocular Microscope (Omax). (**C**) Representative immunocytochemistry images from WT cortical neurons plated on a separate control plate with the same density used in MEA cultures. The dendritic marker MAP2 was stained to show the neuronal processes. Images were acquired on DIV 14 with Zeiss LSM 700 Confocal Microscope.
(TIF)

S2 Fig Neuronal burst activity after AMPA or NBQX treatment. The bursting activity after the treatment of (**A**) AMPA (1 μM, 15 min), (**B**) NBQX (2 μM, 15 min) or vehicle control (ddH_2_O for AMPA and DMSO for NBQX) in WT or *Nedd4-2*^*andi*^ cortical neuron cultures were measured. Three criteria were used to analyze electrode burst activity: burst duration (left), number of spikes per burst (middle) and the interburst interval (right). The data were plotted as “after treatment” normalized to “before treatment”. For the quantification, a two-way ANOVA with post-hoc Tukey test was used. Data are represented as mean ± SEM. The comparison between treatments or genotypes is described with *p<0.05, **p<0.01, ns: non-significant. No significant interaction between treatment and genotype was detected in these data; p>0.05.
(TIF)

S3 Fig Synchrony index after AMPA or NBQX treatment. The bursting activity after the treatment of (**A**) AMPA (1 μM, 15 min), (**B**) NBQX (2 μM, 15 min) or vehicle control (ddH_2_O for AMPA and DMSO for NBQX) in WT or *Nedd4-2*^*andi*^ cortical neuron cultures were measured. The data were plotted as “after treatment” normalized to “before treatment”. For the quantification, a two-way ANOVA with post-hoc Tukey test was used. Data are represented as mean ± SEM. No significance was detected between treatments or genotypes (ns: non-significant). No significant interaction between treatment and genotype was detected in these data either; p>0.05.
(TIF)

S4 Fig HEK cells do not express detectable level of Nedd4-2 or GluA1. Western blots of Nedd4-2, GluA1, and Actin from WT cortical neuron culture lysate or HEK cell lysate. The experiment was repeated 3 times.
(TIF)

S5 Fig K868 of GluA1 is the major residue ubiquitinated by Nedd4-2. Western blots of Ubiquitin (Ub) or GluA1 after GluA1 immunoprecipitation from HEK cells transfected with WT or mutant Nedd4-2s along with WT- or K868R-GluA1 for 48 hours. Quantification of ubiquitinated GluA1 by the entire area of smear from 100–250 kDa is shown on the right (n = 4). Student *t*-test was used for comparison between WT- and K868R-GluA1 in each group. One-way ANOVA with post-hoc Tukey test was used for comparison between different Nedd4-2s.
(TIF)

S6 Fig Three epilepsy-associated missense mutations of Nedd4-2 reduce GluA1 degradation. Representative western blots of GluA1, Nedd4-2, and Actin from HEK cells transfected with GluA1 along with WT or mutant Nedd4-2s after 0- and 8-hr MG132 (10 μM) treatment are shown. The data were plotted as “after treatment” normalized to “before treatment”. The quantification of GluA1 and Nedd4-2 levels after MG132 treatment are on the right (n = 4, one-way ANOVA with post-hoc Tukey test). Data are represented as mean ± SEM with *p<0.05, **p<0.01.
(TIF)

S7 Fig A schematic structure of Nedd4-2 and the location of the three residues of epilepsy-associated missense mutations. All three residues of epilepsy-associated mutations are located on or near one of the protein-protein interaction domains (WW domains).
(TIF)

S8 Fig Three epilepsy-associated missense mutations of Nedd4-2 exhibit reduced interaction with 14-3-3. Western blots of Nedd4-2 and 14-3-3 (pan) after using recombinant 14-3-3ε to immunoprecipitate Nedd4-2 from the lysates of HEK cells transfected with WT or mutant Nedd4-2s for 48 hours. Right before the washing, 5% of total protein mixture was obtained and used as input control shown on the bottom. Quantification is on the right (n = 3, one-way ANOVA with post-hoc Tukey test). Data are represented as mean ± SEM with *p<0.05.
(TIF)

S9 Fig Nedd4-2 does not regulate the level of 14-3-3. Western blots of Nedd4-2, 14-3-3, and Actin from WT or *Nedd4-2*^*andi*^ brain lysates. Quantification is performed using Student *t*-test (n = 4). Data are represented as mean ± SEM with ns: non-significant.
(TIF)

S10 Fig Overexpression of WT or mutant Nedd4-2 in WT cortical neuron cultures does not affect endogenous AMPAR level. (**A**) Western blots of GluA1, N-cadherin, HA-Nedd4-2, and Actin from WT cultures lentivirally transduced with or without WT or mutant Nedd4-2s for 5 days starting at DIV 9. Proteins from total lysate or after surface biotinylation were as indicated. Quantification of (**B**) total and (**C**) surface GluA1 are on the right (n = 3, one-way ANOVA with post-hoc Tukey test). Data are represented as mean ± SEM. No significance was detected between any two transductions.
(TIF)

## References

[1] Grant SG. Synaptopathies: diseases of the synaptome. Current opinion in neurobiology. 2012;22(3):522–9. Epub 2012/03/14. 10.1016/j.conb.2012.02.002 22409856

[2] Zoghbi HY, Bear MF. Synaptic dysfunction in neurodevelopmental disorders associated with autism and intellectual disabilities. Cold Spring Harbor perspectives in biology. 2012;4(3). Epub 2012/01/20. PubMed Central PMCID: PMCPmc3282414. 10.1101/cshperspect.a009886 PMC3282414 22258914

[3] Allen AS, Berkovic SF, Cossette P, Delanty N, Dlugos D, Eichler EE, et al De novo mutations in epileptic encephalopathies. Nature. 2013;501(7466):217–21. Epub 2013/08/13. PubMed Central PMCID: PMCPMC3773011. 10.1038/nature12439 23934111 PMC3773011

[4] Dibbens LM, Ekberg J, Taylor I, Hodgson BL, Conroy SJ, Lensink IL, et al NEDD4-2 as a potential candidate susceptibility gene for epileptic photosensitivity. Genes, brain, and behavior. 2007;6(8):750–5. Epub 2007/03/03. 10.1111/j.1601-183X.2007.00305.x 17331106

[5] Vanli-Yavuz EN, Ozdemir O, Demirkan A, Catal S, Bebek N, Ozbek U, et al Investigation of the possible association of NEDD4-2 (NEDD4L) gene with idiopathic photosensitive epilepsy. Acta neurologica Belgica. 2014;115(3):241–5. Epub 2014/12/30. 10.1007/s13760-014-0412-x 25542253

[6] Wu L, Peng J, Kong H, Yang P, He F, Deng X, et al The role of ubiquitin/Nedd4-2 in the pathogenesis of mesial temporal lobe epilepsy. Physiology & behavior. 2015;143:104–12. Epub 2015/02/24. 25700894 10.1016/j.physbeh.2015.02.026

[7] Yang B, Kumar S. Nedd4 and Nedd4-2: closely related ubiquitin-protein ligases with distinct physiological functions. Cell death and differentiation. 2010;17(1):68–77. Epub 2009/06/27. PubMed Central PMCID: PMCPmc2818775. 10.1038/cdd.2009.84 19557014 PMC2818775

[8] Itani OA, Stokes JB, Thomas CP. Nedd4-2 isoforms differentially associate with ENaC and regulate its activity. American journal of physiology Renal physiology. 2005;289(2):F334–46. Epub 2005/04/09. 10.1152/ajprenal.00394.2004 15814530

[9] Ekberg JA, Boase NA, Rychkov G, Manning J, Poronnik P, Kumar S. Nedd4-2 (NEDD4L) controls intracellular Na(+)-mediated activity of voltage-gated sodium channels in primary cortical neurons. The Biochemical journal. 2014;457(1):27–31. Epub 2013/10/25. 10.1042/BJ20131275 24152020

[10] Ekberg J, Schuetz F, Boase NA, Conroy SJ, Manning J, Kumar S, et al Regulation of the voltage-gated K(+) channels KCNQ2/3 and KCNQ3/5 by ubiquitination. Novel role for Nedd4-2. The Journal of biological chemistry. 2007;282(16):12135–42. Epub 2007/02/27. 10.1074/jbc.M609385200 17322297

[11] Goel P, Manning JA, Kumar S. NEDD4-2 (NEDD4L): the ubiquitin ligase for multiple membrane proteins. Gene. 2015;557(1):1–10. Epub 2014/11/30. 10.1016/j.gene.2014.11.051 25433090 PMC6636357

[12] Schuetz F, Kumar S, Poronnik P, Adams DJ. Regulation of the voltage-gated K(+) channels KCNQ2/3 and KCNQ3/5 by serum- and glucocorticoid-regulated kinase-1. American journal of physiology Cell physiology. 2008;295(1):C73–80. Epub 2008/05/09. 10.1152/ajpcell.00146.2008 18463232

[13] Georgieva MV, de Pablo Y, Sanchis D, Comella JX, Llovera M. Ubiquitination of TrkA by Nedd4-2 regulates receptor lysosomal targeting and mediates receptor signaling. Journal of neurochemistry. 2011;117(3):479–93. Epub 2011/02/22. 10.1111/j.1471-4159.2011.07218.x 21332718

[14] Yu T, Calvo L, Anta B, Lopez-Benito S, Lopez-Bellido R, Vicente-Garcia C, et al In vivo regulation of NGF-mediated functions by Nedd4-2 ubiquitination of TrkA. The Journal of neuroscience: the official journal of the Society for Neuroscience. 2014;34(17):6098–106. Epub 2014/04/25. PubMed Central PMCID: PMCPmc3996226. 24760869 10.1523/JNEUROSCI.4271-13.2014 PMC3996226

[15] Jewett KA, Zhu J, Tsai NP. The tumor suppressor p53 guides glua1 homeostasis through Nedd4-2 during chrnoic elevation of neuronal activity. J Neurochem. 2015;135(2):226–33. 10.1111/jnc.13271 26250624

[16] Jewett KA, Christian CA, Bacos JT, Lee KY, Zhu J, Tsai NP. Feedback modulation of neural network synchrony and seizure susceptibility by Mdm2-p53-Nedd4-2 signaling. Molecular brain. 2016;9(1):32. Epub 2016/03/24. PubMed Central PMCID: PMCPmc4802718. 27000207 10.1186/s13041-016-0214-6 PMC4802718

[17] Bowie D. Redefining the classification of AMPA-selective ionotropic glutamate receptors. The Journal of physiology. 2012;590(1):49–61. Epub 2011/11/23. PubMed Central PMCID: PMCPmc3300045. 10.1113/jphysiol.2011.221689 22106175 PMC3300045

[18] Hall BJ, Ghosh A. Regulation of AMPA receptor recruitment at developing synapses. Trends in neurosciences. 2008;31(2):82–9. Epub 2008/01/19. 10.1016/j.tins.2007.11.010 18201773

[19] Nakagawa T. The biochemistry, ultrastructure, and subunit assembly mechanism of AMPA receptors. Molecular neurobiology. 2010;42(3):161–84. Epub 2010/11/17. PubMed Central PMCID: PMCPmc2992128. 10.1007/s12035-010-8149-x 21080238 PMC2992128

[20] Fu AK, Hung KW, Fu WY, Shen C, Chen Y, Xia J, et al APC(Cdh1) mediates EphA4-dependent downregulation of AMPA receptors in homeostatic plasticity. Nature neuroscience. 2011;14(2):181–9. Epub 2010/12/28. 10.1038/nn.2715 21186356

[21] Widagdo J, Chai YJ, Ridder MC, Chau YQ, Johnson RC, Sah P, et al Activity-dependent ubiquitination of GluA1 and GluA2 regulates AMPA receptor intracellular sorting and degradation. Cell reports. 2015;10(5):783–95. Epub 2015/02/11. PubMed Central PMCID: PMCPmc4524782. 10.1016/j.celrep.2015.01.015 PMC4524782 25660027

[22] Lin A, Hou Q, Jarzylo L, Amato S, Gilbert J, Shang F, et al Nedd4-mediated AMPA receptor ubiquitination regulates receptor turnover and trafficking. Journal of neurochemistry. 2011;119(1):27–39. Epub 2011/02/23. PubMed Central PMCID: PMCPmc3110981. 10.1111/j.1471-4159.2011.07221.x 21338354 PMC3110981

[23] Schwarz LA, Hall BJ, Patrick GN. Activity-dependent ubiquitination of GluA1 mediates a distinct AMPA receptor endocytosis and sorting pathway. The Journal of neuroscience: the official journal of the Society for Neuroscience. 2010;30(49):16718–29. Epub 2010/12/15. PubMed Central PMCID: PMCPmc3079366. 21148011 10.1523/JNEUROSCI.3686-10.2010 PMC3079366

[24] Malenka RC. Synaptic plasticity and AMPA receptor trafficking. Annals of the New York Academy of Sciences. 2003;1003:1–11. Epub 2003/12/20. 14684431 10.1196/annals.1300.001

[25] Anggono V, Koc-Schmitz Y, Widagdo J, Kormann J, Quan A, Chen CM, et al PICK1 interacts with PACSIN to regulate AMPA receptor internalization and cerebellar long-term depression. Proceedings of the National Academy of Sciences of the United States of America. 2013;110(34):13976–81. Epub 2013/08/07. PubMed Central PMCID: PMCPmc3752261. 10.1073/pnas.1312467110 23918399 PMC3752261

[26] Glebov OO, Tigaret CM, Mellor JR, Henley JM. Clathrin-independent trafficking of AMPA receptors. The Journal of neuroscience: the official journal of the Society for Neuroscience. 2015;35(12):4830–6. Epub 2015/03/27. 25810514 10.1523/JNEUROSCI.3571-14.2015 PMC4389590

[27] McCartney AJ, Zolov SN, Kauffman EJ, Zhang Y, Strunk BS, Weisman LS, et al Activity-dependent PI(3,5)P2 synthesis controls AMPA receptor trafficking during synaptic depression. Proceedings of the National Academy of Sciences of the United States of America. 2014;111(45):E4896–905. Epub 2014/10/31. PubMed Central PMCID: PMCPmc4234577. 10.1073/pnas.1411117111 25355904 PMC4234577

[28] Henley JM, Barker EA, Glebov OO. Routes, destinations and delays: recent advances in AMPA receptor trafficking. Trends in neurosciences. 2011;34(5):258–68. Epub 2011/03/23. PubMed Central PMCID: PMCPmc3314507. 10.1016/j.tins.2011.02.004 21420743 PMC3314507

[29] Lu W, Bushong EA, Shih TP, Ellisman MH, Nicoll RA. The cell-autonomous role of excitatory synaptic transmission in the regulation of neuronal structure and function. Neuron. 2013;78(3):433–9. Epub 2013/05/15. PubMed Central PMCID: PMCPmc3666354. 10.1016/j.neuron.2013.02.030 23664612 PMC3666354

[30] Zamanillo D, Sprengel R, Hvalby O, Jensen V, Burnashev N, Rozov A, et al Importance of AMPA receptors for hippocampal synaptic plasticity but not for spatial learning. Science (New York, NY). 1999;284(5421):1805–11. Epub 1999/06/12. 10.1126/science.284.5421.1805 10364547

[31] Zhang L, Schessl J, Werner M, Bonnemann C, Xiong G, Mojsilovic-Petrovic J, et al Role of GluR1 in activity-dependent motor system development. The Journal of neuroscience: the official journal of the Society for Neuroscience. 2008;28(40):9953–68. Epub 2008/10/03. PubMed Central PMCID: PMCPmc3844744. 18829953 10.1523/JNEUROSCI.0880-08.2008 PMC3844744

[32] Rogawski MA, Donevan SD. AMPA receptors in epilepsy and as targets for antiepileptic drugs. Advances in neurology. 1999;79:947–63. Epub 1999/10/09. 10514878

[33] Bateup HS, Denefrio CL, Johnson CA, Saulnier JL, Sabatini BL. Temporal dynamics of a homeostatic pathway controlling neural network activity. Frontiers in molecular neuroscience. 2013;6:28 Epub 2013/09/26. PubMed Central PMCID: PMCPMC3776619. 10.3389/fnmol.2013.00028 24065881 PMC3776619

[34] Boase NA, Rychkov GY, Townley SL, Dinudom A, Candi E, Voss AK, et al Respiratory distress and perinatal lethality in Nedd4-2-deficient mice. Nature communications. 2011;2:287 Epub 2011/04/21. PubMed Central PMCID: PMCPMC3104547. 10.1038/ncomms1284 21505443 PMC3104547

[35] Keefer EW, Gramowski A, Gross GW. NMDA receptor-dependent periodic oscillations in cultured spinal cord networks. Journal of neurophysiology. 2001;86(6):3030–42. Epub 2001/12/04. 11731558 10.1152/jn.2001.86.6.3030

[36] Wu G, Lu ZH, Wang J, Wang Y, Xie X, Meyenhofer MF, et al Enhanced susceptibility to kainate-induced seizures, neuronal apoptosis, and death in mice lacking gangliotetraose gangliosides: protection with LIGA 20, a membrane-permeant analog of GM1. The Journal of neuroscience: the official journal of the Society for Neuroscience. 2005;25(47):11014–22. Epub 2005/11/25. 16306414 10.1523/JNEUROSCI.3635-05.2005 PMC6725874

[37] Brusa R, Zimmermann F, Koh DS, Feldmeyer D, Gass P, Seeburg PH, et al Early-onset epilepsy and postnatal lethality associated with an editing-deficient GluR-B allele in mice. Science (New York, NY). 1995;270(5242):1677–80. Epub 1995/12/08. 10.1126/science.270.5242.1677 7502080

[38] Chapman AG. Glutamate and epilepsy. The Journal of nutrition. 2000;130(4S Suppl):1043s–5s. Epub 2000/03/29. 10736378 10.1093/jn/130.4.1043S

[39] Rajasekaran K, Joshi S, Kozhemyakin M, Todorovic MS, Kowalski S, Balint C, et al Receptor trafficking hypothesis revisited: plasticity of AMPA receptors during established status epilepticus. Epilepsia. 2013;54 Suppl 6:14–6. Epub 2013/09/06. 10.1111/epi.12266 24001062

[40] Rajasekaran K, Todorovic M, Kapur J. Calcium-permeable AMPA receptors are expressed in a rodent model of status epilepticus. Annals of neurology. 2012;72(1):91–102. Epub 2012/07/26. PubMed Central PMCID: PMCPmc3408623. 10.1002/ana.23570 22829271 PMC3408623

[41] Bruce MC, Kanelis V, Fouladkou F, Debonneville A, Staub O, Rotin D. Regulation of Nedd4-2 self-ubiquitination and stability by a PY motif located within its HECT-domain. The Biochemical journal. 2008;415(1):155–63. Epub 2008/05/24. 10.1042/BJ20071708 18498246

[42] Cui Z, Zhang S. Regulation of the human ether-a-go-go-related gene (hERG) channel by Rab4 protein through neural precursor cell-expressed developmentally down-regulated protein 4–2 (Nedd4-2). The Journal of biological chemistry. 2013;288(30):21876–86. Epub 2013/06/25. PubMed Central PMCID: PMCPmc3724643. 10.1074/jbc.M113.461715 23792956 PMC3724643

[43] Chandran S, Li H, Dong W, Krasinska K, Adams C, Alexandrova L, et al Neural precursor cell-expressed developmentally down-regulated protein 4–2 (Nedd4-2) regulation by 14-3-3 protein binding at canonical serum and glucocorticoid kinase 1 (SGK1) phosphorylation sites. The Journal of biological chemistry. 2011;286(43):37830–40. Epub 2011/09/09. PubMed Central PMCID: PMCPmc3199524. 10.1074/jbc.M111.293233 21900244 PMC3199524

[44] Liang X, Peters KW, Butterworth MB, Frizzell RA. 14-3-3 isoforms are induced by aldosterone and participate in its regulation of epithelial sodium channels. The Journal of biological chemistry. 2006;281(24):16323–32. Epub 2006/04/15. 10.1074/jbc.M601360200 16613846

[45] Nagaki K, Yamamura H, Shimada S, Saito T, Hisanaga S, Taoka M, et al 14-3-3 Mediates phosphorylation-dependent inhibition of the interaction between the ubiquitin E3 ligase Nedd4-2 and epithelial Na+ channels. Biochemistry. 2006;45(21):6733–40. Epub 2006/05/24. 10.1021/bi052640q 16716084

[46] Liang X, Butterworth MB, Peters KW, Walker WH, Frizzell RA. An obligatory heterodimer of 14-3-3beta and 14-3-3epsilon is required for aldosterone regulation of the epithelial sodium channel. The Journal of biological chemistry. 2008;283(41):27418–25. Epub 2008/08/09. PubMed Central PMCID: PMCPmc2562081. 10.1074/jbc.M803687200 18687683 PMC2562081

[47] Dong S, Kang S, Lonial S, Khoury HJ, Viallet J, Chen J. Targeting 14-3-3 sensitizes native and mutant BCR-ABL to inhibition with U0126, rapamycin and Bcl-2 inhibitor GX15-070. Leukemia. 2008;22(3):572–7. Epub 2007/12/15. PubMed Central PMCID: PMCPmc2396184. 10.1038/sj.leu.2405064 18079735 PMC2396184

[48] Du Y, Khuri FR, Fu H. A homogenous luminescent proximity assay for 14-3-3 interactions with both phosphorylated and nonphosphorylated client peptides. Current chemical genomics. 2008;2:40–7. Epub 2008/01/01. PubMed Central PMCID: PMCPmc2803432. 10.2174/1875397300802010040 20161842 PMC2803432

[49] Fujita N, Sato S, Tsuruo T. Phosphorylation of p27Kip1 at threonine 198 by p90 ribosomal protein S6 kinases promotes its binding to 14-3-3 and cytoplasmic localization. The Journal of biological chemistry. 2003;278(49):49254–60. Epub 2003/09/25. 10.1074/jbc.M306614200 14504289

[50] Chang P, Augustin K, Boddum K, Williams S, Sun M, Terschak JA, et al Seizure control by decanoic acid through direct AMPA receptor inhibition. Brain: a journal of neurology. 2015;139(Pt 2):431–43. Epub 2015/11/27. 26608744 10.1093/brain/awv325 PMC4805082

[51] Rogawski MA. AMPA receptors as a molecular target in epilepsy therapy. Acta neurologica Scandinavica Supplementum. 2013;(197):9–18. Epub 2013/03/27. PubMed Central PMCID: PMCPmc4506648. 10.1111/ane.12099 23480151 PMC4506648

[52] Rogawski MA, Hanada T. Preclinical pharmacology of perampanel, a selective non-competitive AMPA receptor antagonist. Acta neurologica Scandinavica Supplementum. 2013;(197):19–24. Epub 2013/03/27. PubMed Central PMCID: PMCPmc4506647. 10.1111/ane.12100 23480152 PMC4506647

[53] Garrone NF, Blazer-Yost BL, Weiss RB, Lalouel JM, Rohrwasser A. A human polymorphism affects NEDD4L subcellular targeting by leading to two isoforms that contain or lack a C2 domain. BMC cell biology. 2009;10:26 Epub 2009/04/15. PubMed Central PMCID: PMCPmc2678989. 10.1186/1471-2121-10-26 19364400 PMC2678989

[54] Dahlberg J, Nilsson LO, von Wowern F, Melander O. Polymorphism in NEDD4L is associated with increased salt sensitivity, reduced levels of P-renin and increased levels of Nt-proANP. PloS one. 2007;2(5):e432 Epub 2007/05/10. PubMed Central PMCID: PMCPmc1855992. 10.1371/journal.pone.0000432 17487281 PMC1855992

[55] Zhu JJ, Esteban JA, Hayashi Y, Malinow R. Postnatal synaptic potentiation: delivery of GluR4-containing AMPA receptors by spontaneous activity. Nature neuroscience. 2000;3(11):1098–106. Epub 2000/10/19. 10.1038/80614 11036266

[56] Davis GW. Homeostatic signaling and the stabilization of neural function. Neuron. 2013;80(3):718–28. Epub 2013/11/05. PubMed Central PMCID: PMCPmc3856728. 10.1016/j.neuron.2013.09.044 24183022 PMC3856728

[57] Briz V, Liu Y, Zhu G, Bi X, Baudry M. A novel form of synaptic plasticity in field CA3 of hippocampus requires GPER1 activation and BDNF release. The Journal of cell biology. 2015;210(7):1225–37. Epub 2015/09/24. PubMed Central PMCID: PMCPmc4586750. 10.1083/jcb.201504092 26391661 PMC4586750

[58] Tsai NP, Wilkerson JR, Guo W, Maksimova MA, DeMartino GN, Cowan CW, et al Multiple autism-linked genes mediate synapse elimination via proteasomal degradation of a synaptic scaffold PSD-95. Cell. 2012;151(7):1581–94. Epub 2012/12/25. PubMed Central PMCID: PMCPmc3530171. 10.1016/j.cell.2012.11.040 23260144 PMC3530171

[59] Kim E, Wang B, Sastry N, Masliah E, Nelson PT, Cai H, et al NEDD4-mediated HSF1 degradation underlies alpha-synucleinopathy. Human molecular genetics. 2016;25(2):211–22. Epub 2015/10/28. PubMed Central PMCID: PMCPmc4706110. 10.1093/hmg/ddv445 26503960 PMC4706110

[60] Tardiff DF, Jui NT, Khurana V, Tambe MA, Thompson ML, Chung CY, et al Yeast reveal a "druggable" Rsp5/Nedd4 network that ameliorates alpha-synuclein toxicity in neurons. Science (New York, NY). 2013;342(6161):979–83. Epub 2013/10/26. PubMed Central PMCID: PMCPmc3993916. 10.1126/science.1245321 PMC3993916 24158909

[61] Tofaris GK, Kim HT, Hourez R, Jung JW, Kim KP, Goldberg AL. Ubiquitin ligase Nedd4 promotes alpha-synuclein degradation by the endosomal-lysosomal pathway. Proceedings of the National Academy of Sciences of the United States of America. 2011;108(41):17004–9. Epub 2011/09/29 PubMed Central PMCID: PMCPmc3193191. 10.1073/pnas.1109356108 21953697 PMC3193191

[62] Barker-Gibb AL, Dougherty KD, Einheber S, Drake CT, Milner TA. Hippocampal tyrosine kinase A receptors are restricted primarily to presynaptic vesicle clusters. The Journal of comparative neurology. 2001;430(2):182–99. Epub 2001/01/03. 11135255 10.1002/1096-9861(20010205)430:2<182::aid-cne1024>3.0.co;2-q

[63] Woelk T, Oldrini B, Maspero E, Confalonieri S, Cavallaro E, Di Fiore PP, et al Molecular mechanisms of coupled monoubiquitination. Nat Cell Biol. 2006;8(11):1246–54. Epub 2006/10/03. 10.1038/ncb1484 17013377

[64] McConnell ER, McClain MA, Ross J, Lefew WR, Shafer TJ. Evaluation of multi-well microelectrode arrays for neurotoxicity screening using a chemical training set. Neurotoxicology. 2012;33(5):1048–57. Epub 2012/06/02. PubMed Central PMCID: PMCPmc3721981. 10.1016/j.neuro.2012.05.001 22652317 PMC3721981

[65] Jewett KA, Taishi P, Sengupta P, Roy S, Davis CJ, Krueger JM. Tumor necrosis factor enhances the sleep-like state and electrical stimulation induces a wake-like state in co-cultures of neurons and glia. The European journal of neuroscience. 2015;42(4):2078–90. Epub 2015/06/04. PubMed Central PMCID: PMCPmc4540611. 10.1111/ejn.12968 26036796 PMC4540611

[66] McSweeney KM, Gussow AB, Bradrick SS, Dugger SA, Gelfman S, Wang Q, et al Inhibition of microRNA 128 promotes excitability of cultured cortical neuronal networks. Genome research. 2016;26(10):1411–6. Epub 2016/08/16. PubMed Central PMCID: PMCPmc5052052. 10.1101/gr.199828.115 27516621 PMC5052052

[67] Eggermont JJ. Properties of correlated neural activity clusters in cat auditory cortex resemble those of neural assemblies. Journal of neurophysiology. 2006;96(2):746–64. Epub 2006/07/13. 10.1152/jn.00059.2006 16835364

[68] Tsai NP, Lin YL, Tsui YC, Wei LN. Dual action of epidermal growth factor: extracellular signal-stimulated nuclear-cytoplasmic export and coordinated translation of selected messenger RNA. J Cell Biol. 2010;188(3):325–33. Epub 2010/02/10. PubMed Central PMCID: PMC2819679. 10.1083/jcb.200910083 20142421 PMC2819679

[69] Racine RJ. Modification of seizure activity by electrical stimulation. II. Motor seizure. Electroencephalography and clinical neurophysiology. 1972;32(3):281–94. Epub 1972/03/01. 4110397 10.1016/0013-4694(72)90177-0

[70] Keith DJ, Sanderson JL, Gibson ES, Woolfrey KM, Robertson HR, Olszewski K, et al Palmitoylation of A-kinase anchoring protein 79/150 regulates dendritic endosomal targeting and synaptic plasticity mechanisms. The Journal of neuroscience: the official journal of the Society for Neuroscience. 2012;32(21):7119–36. Epub 2012/05/25. PubMed Central PMCID: PMCPmc3367663. 22623657 10.1523/JNEUROSCI.0784-12.2012 PMC3367663

