# Supplementary figures and images for: Epilepsy-associated gene Nedd4-2 mediates neuronal activity and seizure susceptibility through AMPA receptors
Source: PLoS Genet. 2017 Feb 17;13(2):e1006634. doi: 10.1371/journal.pgen.1006634 (PMC5338825; doi:10.1371/journal.pgen.1006634)

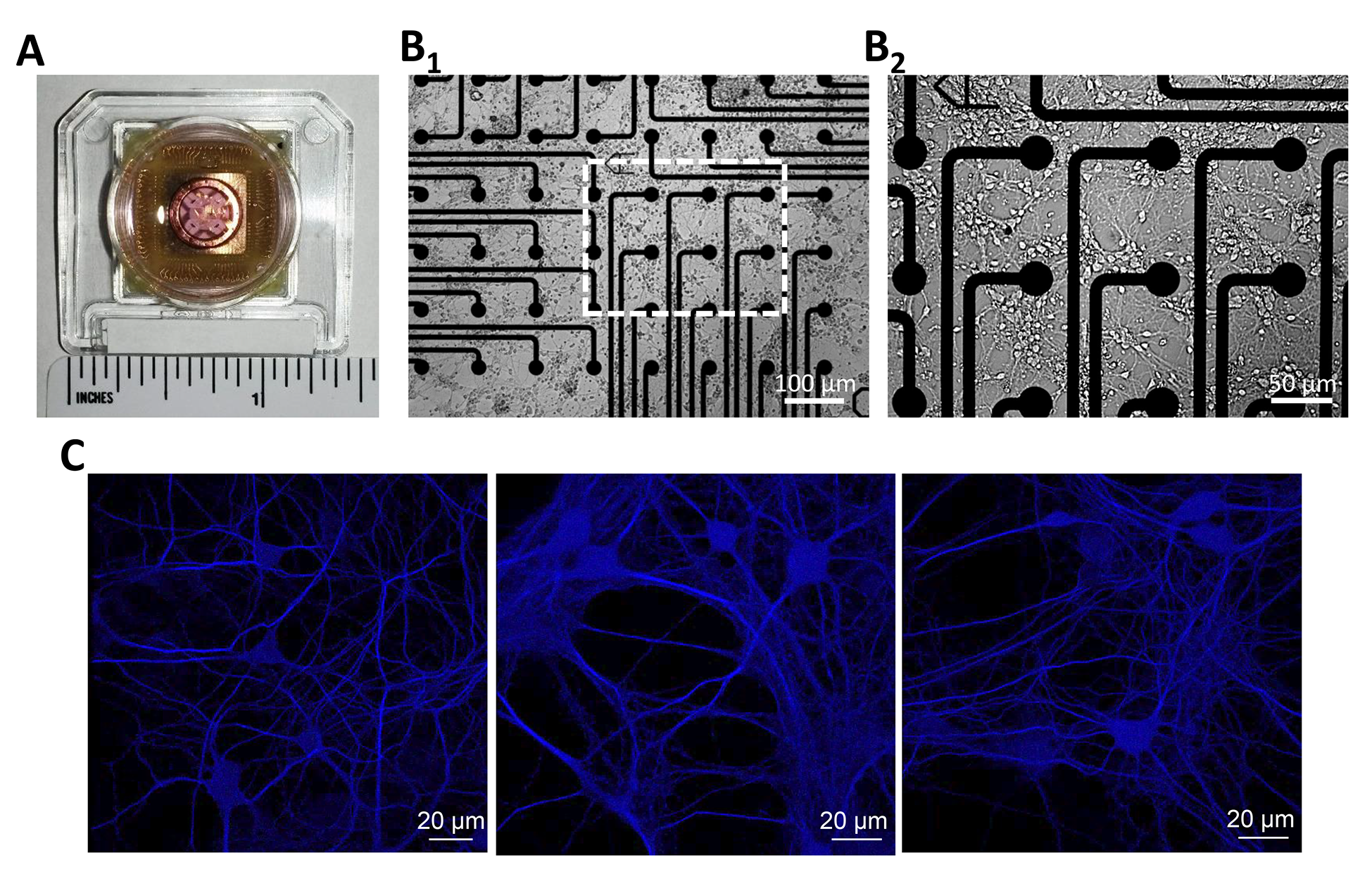

Supplement: S1 Fig — (A) A picture of a multi-electrode array (MEA) dish and (B) a representative image of cultured cortical neurons growing in a MEA dish at DIV 14. A selected area in (B1) is enlarged and shown in (B2). Images were acquired by an EPI-Fluorescence Trinocular Microscope (Omax). (C) Representative immunocytochemistry images from WT cortical neurons plated on a separate control plate with the same density used in MEA cultures. The dendritic marker MAP2 was stained to show the neuronal processes. Images were acquired on DIV 14 with Zeiss LSM 700 Confocal Microscope. (TIF) [file pgen.1006634.s001.tif]

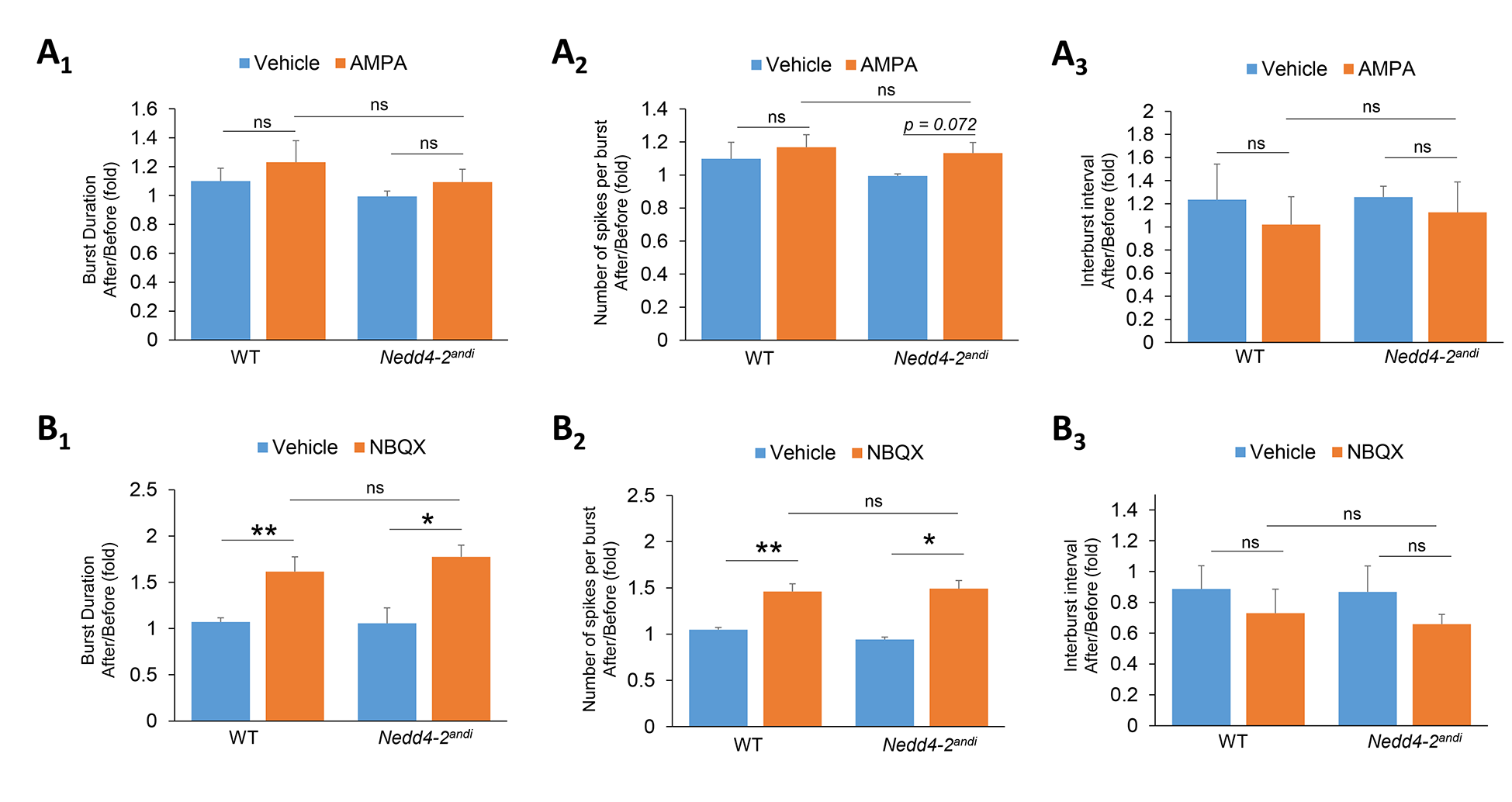

Supplement: S2 Fig — The bursting activity after the treatment of (A) AMPA (1 μM, 15 min), (B) NBQX (2 μM, 15 min) or vehicle control (ddH2O for AMPA and DMSO for NBQX) in WT or Nedd4-2andi cortical neuron cultures were measured. Three criteria were used to analyze electrode burst activity: burst duration (left), number of spikes per burst (middle) and the interburst interval (right). The data were plotted as “after treatment” normalized to “before treatment”. For the quantification, a two-way ANOVA with post-hoc Tukey test was used. Data are represented as mean ± SEM. The comparison between treatments or genotypes is described with *p<0.05, **p<0.01, ns: non-significant. No significant interaction between treatment and genotype was detected in these data; p>0.05. (TIF) [file pgen.1006634.s002.tif]

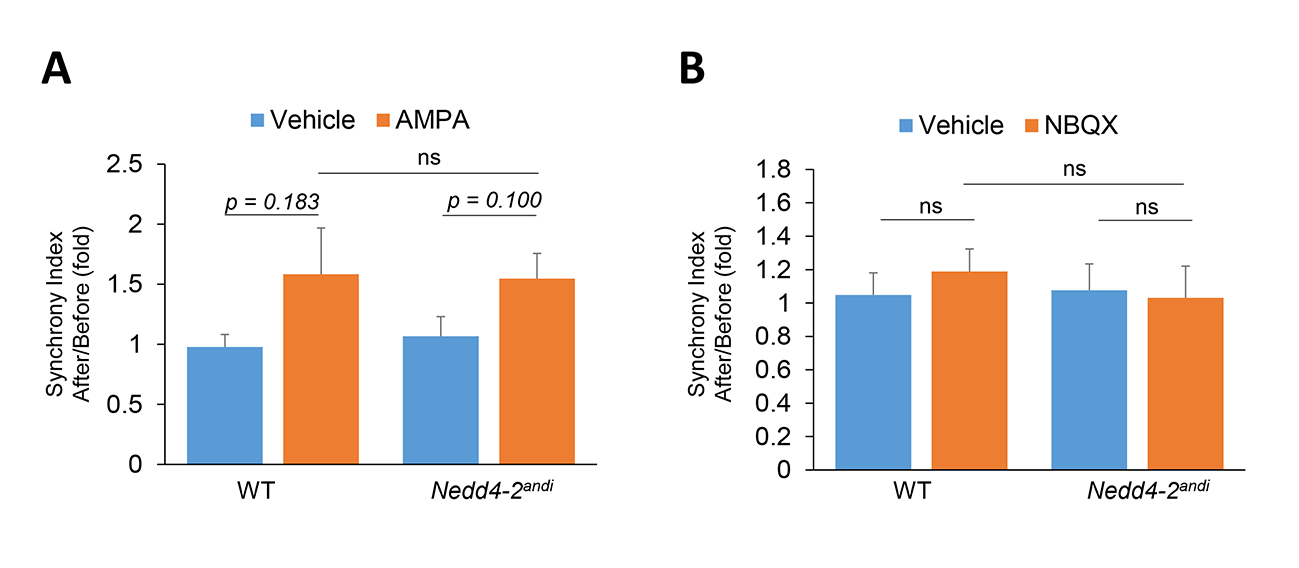

Supplement: S3 Fig — The bursting activity after the treatment of (A) AMPA (1 μM, 15 min), (B) NBQX (2 μM, 15 min) or vehicle control (ddH2O for AMPA and DMSO for NBQX) in WT or Nedd4-2andi cortical neuron cultures were measured. The data were plotted as “after treatment” normalized to “before treatment”. For the quantification, a two-way ANOVA with post-hoc Tukey test was used. Data are represented as mean ± SEM. No significance was detected between treatments or genotypes (ns: non-significant). No significant interaction between treatment and genotype was detected in these data either; p>0.05. (TIF) [file pgen.1006634.s003.tif]

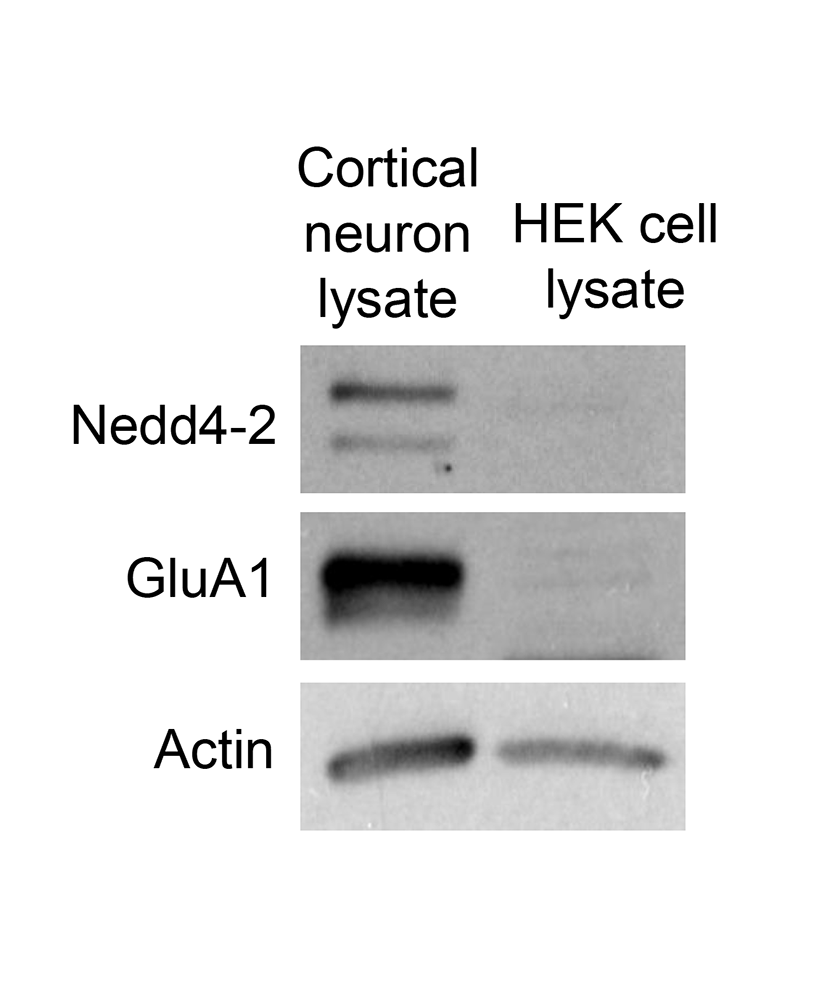

Supplement: S4 Fig — Western blots of Nedd4-2, GluA1, and Actin from WT cortical neuron culture lysate or HEK cell lysate. The experiment was repeated 3 times. (TIF) [file pgen.1006634.s004.tif]

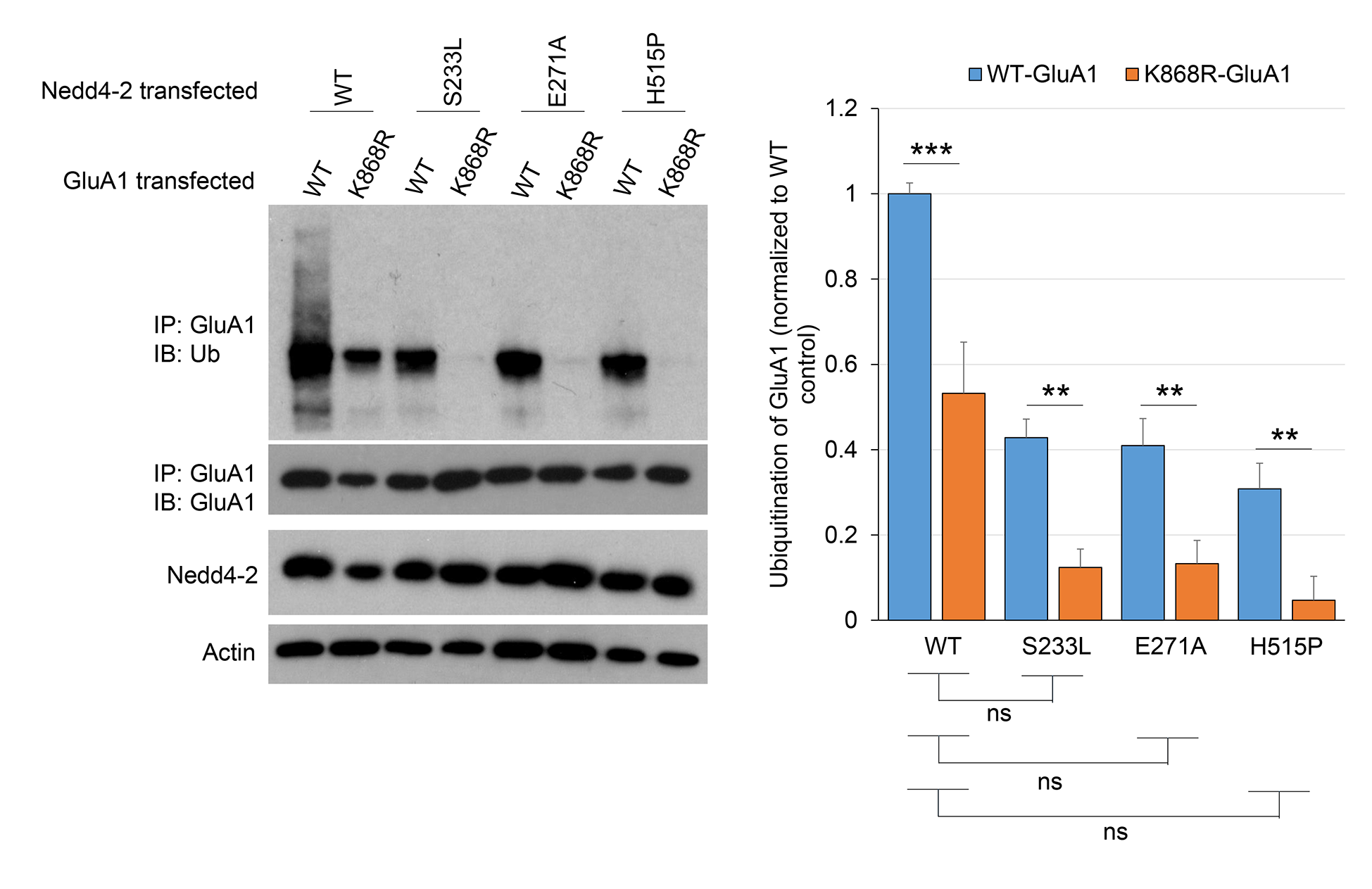

Supplement: S5 Fig — Western blots of Ubiquitin (Ub) or GluA1 after GluA1 immunoprecipitation from HEK cells transfected with WT or mutant Nedd4-2s along with WT- or K868R-GluA1 for 48 hours. Quantification of ubiquitinated GluA1 by the entire area of smear from 100–250 kDa is shown on the right (n = 4). Student t-test was used for comparison between WT- and K868R-GluA1 in each group. One-way ANOVA with post-hoc Tukey test was used for comparison between different Nedd4-2s. (TIF) [file pgen.1006634.s005.tif]

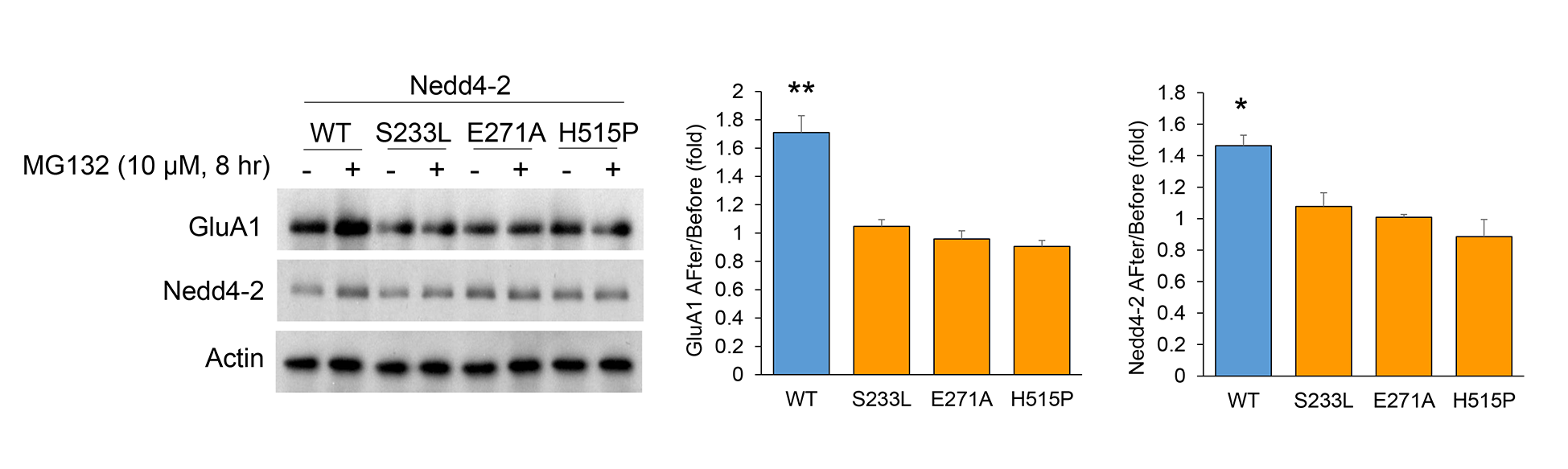

Supplement: S6 Fig — Representative western blots of GluA1, Nedd4-2, and Actin from HEK cells transfected with GluA1 along with WT or mutant Nedd4-2s after 0- and 8-hr MG132 (10 μM) treatment are shown. The data were plotted as “after treatment” normalized to “before treatment”. The quantification of GluA1 and Nedd4-2 levels after MG132 treatment are on the right (n = 4, one-way ANOVA with post-hoc Tukey test). Data are represented as mean ± SEM with *p<0.05, **p<0.01. (TIF) [file pgen.1006634.s006.tif]

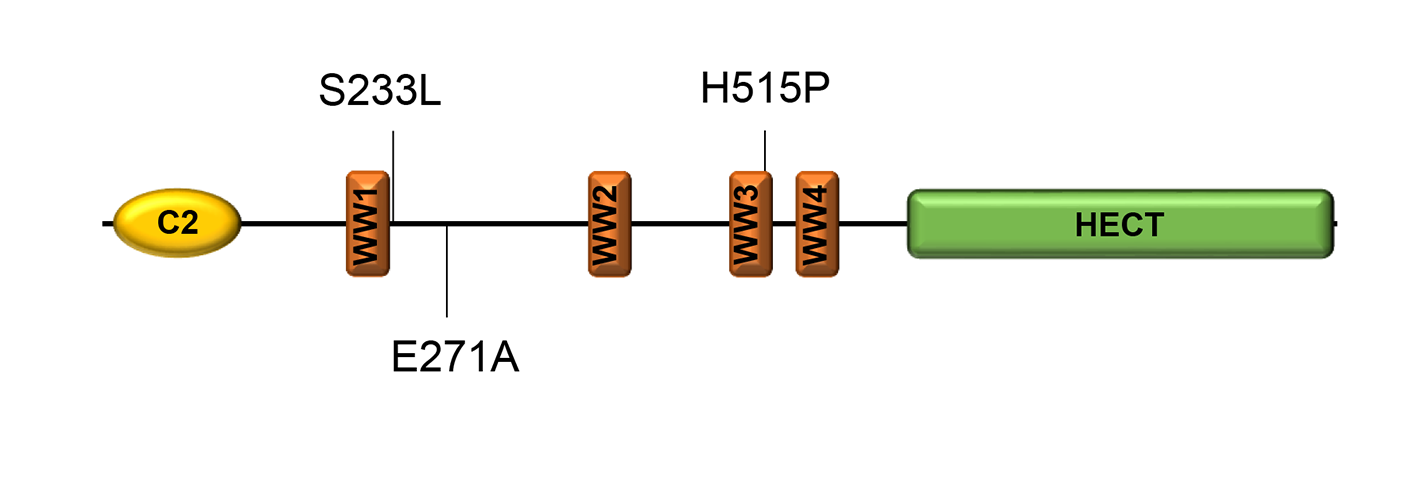

Supplement: S7 Fig — All three residues of epilepsy-associated mutations are located on or near one of the protein-protein interaction domains (WW domains). (TIF) [file pgen.1006634.s007.tif]

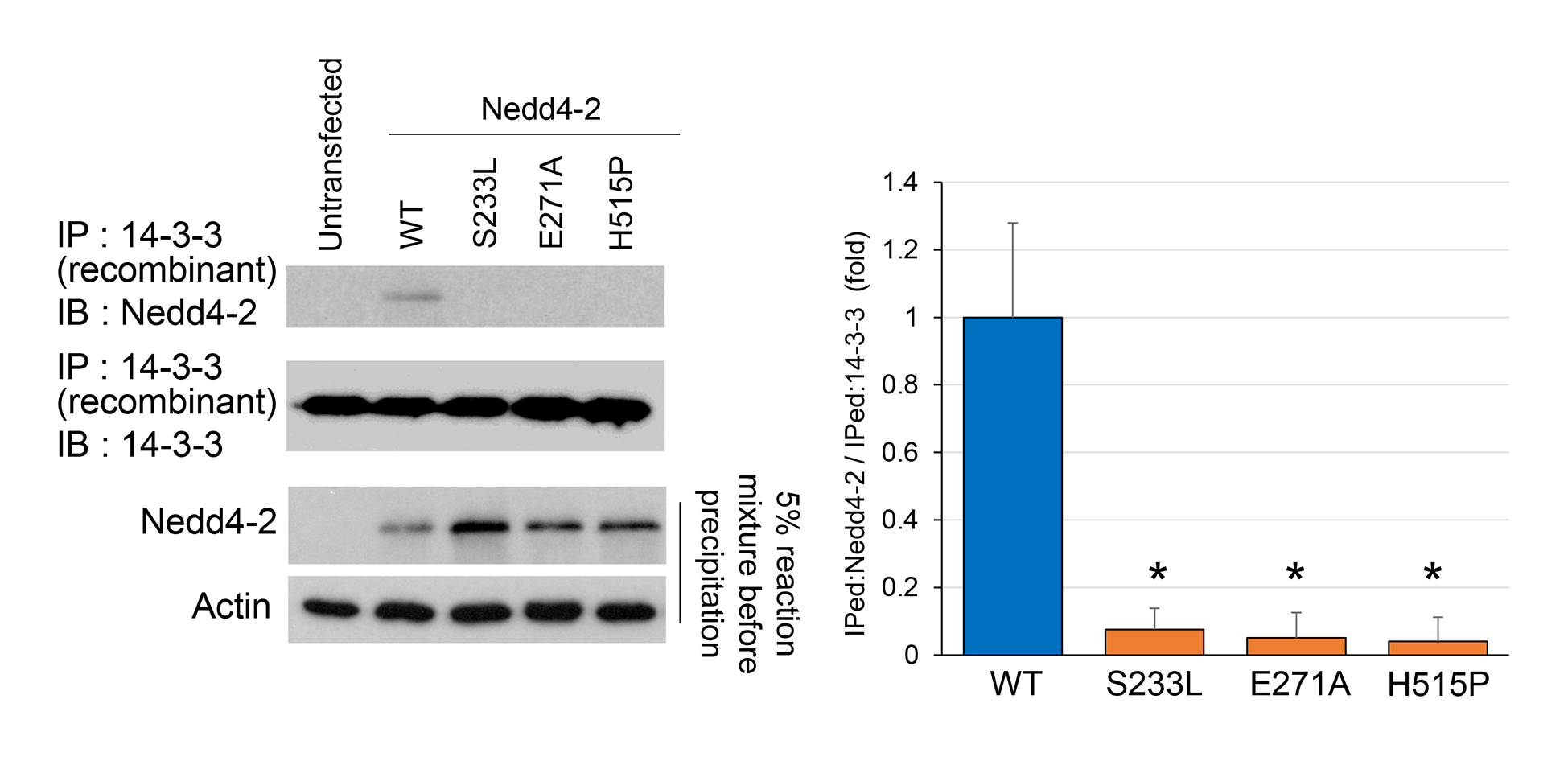

Supplement: S8 Fig — Western blots of Nedd4-2 and 14-3-3 (pan) after using recombinant 14-3-3ε to immunoprecipitate Nedd4-2 from the lysates of HEK cells transfected with WT or mutant Nedd4-2s for 48 hours. Right before the washing, 5% of total protein mixture was obtained and used as input control shown on the bottom. Quantification is on the right (n = 3, one-way ANOVA with post-hoc Tukey test). Data are represented as mean ± SEM with *p<0.05. (TIF) [file pgen.1006634.s008.tif]

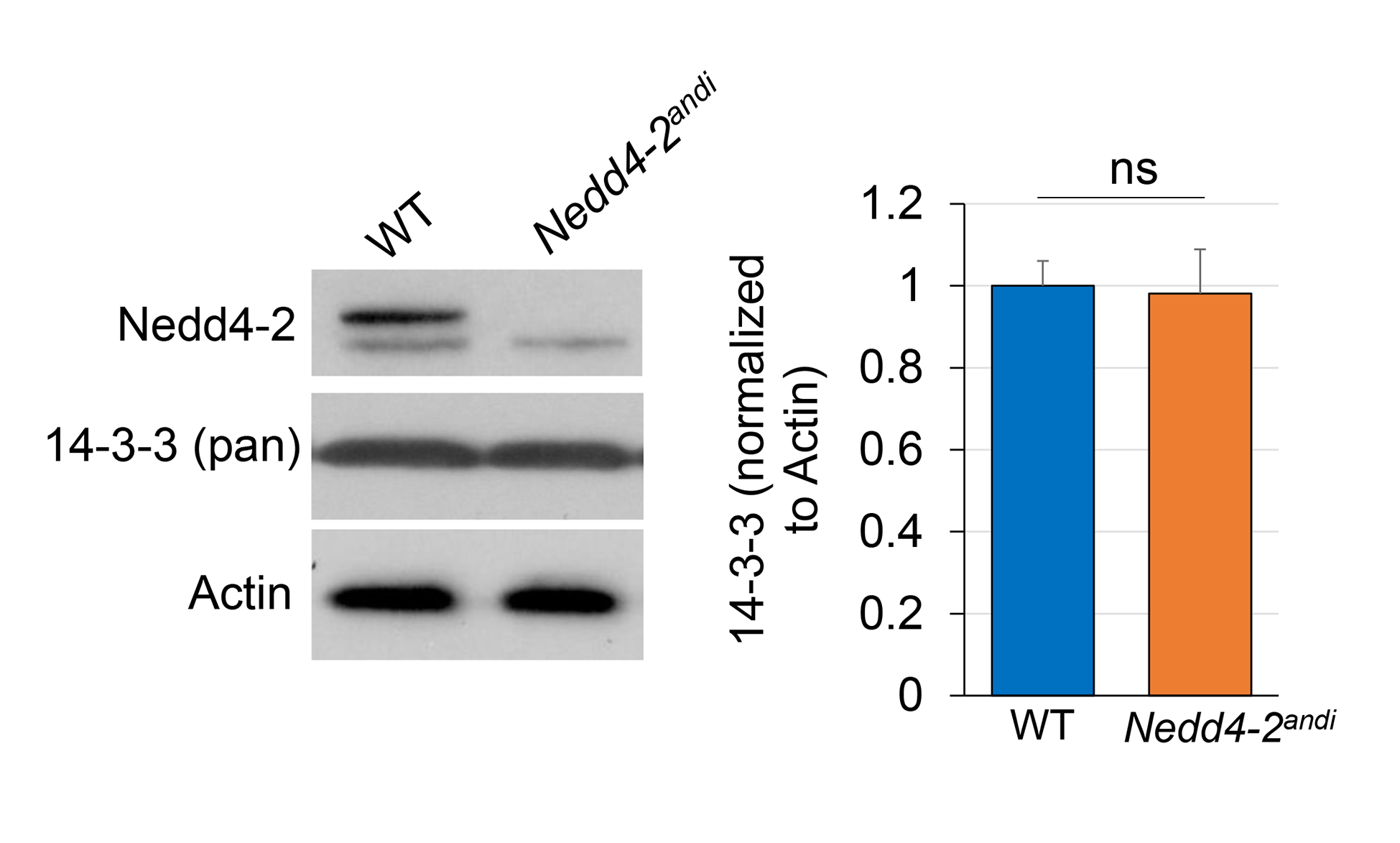

Supplement: S9 Fig — Western blots of Nedd4-2, 14-3-3, and Actin from WT or Nedd4-2andi brain lysates. Quantification is performed using Student t-test (n = 4). Data are represented as mean ± SEM with ns: non-significant. (TIF) [file pgen.1006634.s009.tif]

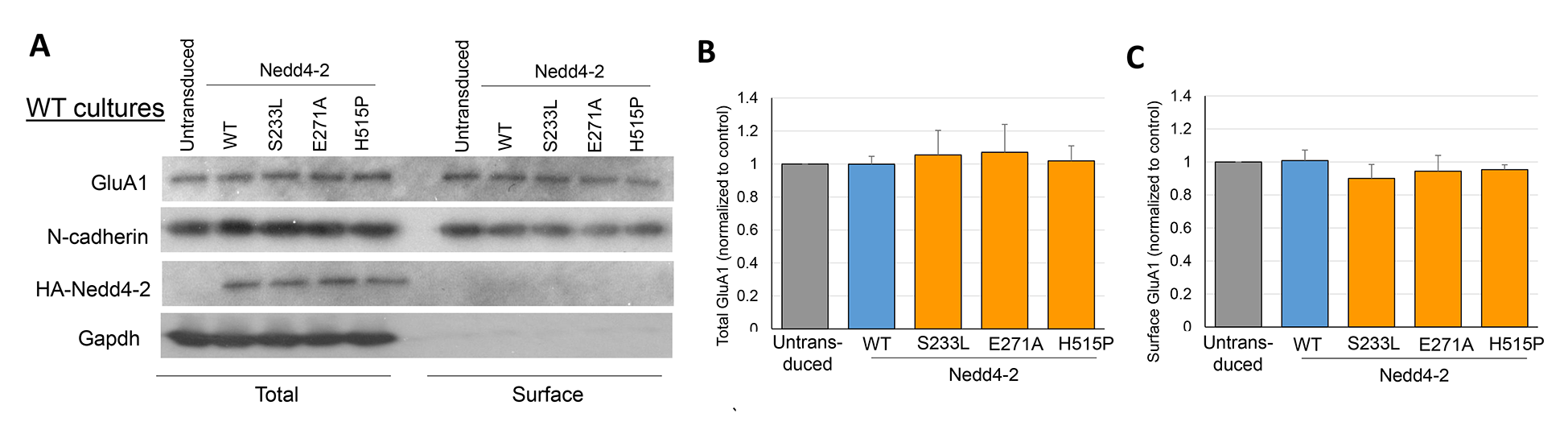

Supplement: S10 Fig — (A) Western blots of GluA1, N-cadherin, HA-Nedd4-2, and Actin from WT cultures lentivirally transduced with or without WT or mutant Nedd4-2s for 5 days starting at DIV 9. Proteins from total lysate or after surface biotinylation were as indicated. Quantification of (B) total and (C) surface GluA1 are on the right (n = 3, one-way ANOVA with post-hoc Tukey test). Data are represented as mean ± SEM. No significance was detected between any two transductions. (TIF) [file pgen.1006634.s010.tif]
